# Supplementary material for: The effect of uncertainty on prediction error in the action perception loop
Source: Cognition. 2021 May;210:104598. doi: 10.1016/j.cognition.2021.104598 (PMC8085733; doi:10.1016/j.cognition.2021.104598)
Supplement: Supplementary material — See supplementary materials document for further methodological details including definitions of policies and the biased-nearest-object method for hypothesis definition. This document also contains copies of all statistical models, including some that were referenced but not reported in the main text. [file mmc1.docx]

Supplementary Material

Table of Contents

[Policy Definitions: 2](#_Toc50734678)

[Horizontal 2](#_Toc50734679)

[Vertical 2](#_Toc50734680)

[Perpendicular-Cardinal 2](#_Toc50734681)

[Non-Cardinal 2](#_Toc50734682)

[Hesitant-Straight 2](#_Toc50734683)

[Circle 3](#_Toc50734684)

[Biased-nearest-object Method for Hypothesis Definition: 3](#_Toc50734685)

[Interaction Results for Movement and Strategy Variables: 3](#_Toc50734686)

[Speed 3](#_Toc50734687)

[Turn Count 3](#_Toc50734688)

[Hypothesis Switch Count 4](#_Toc50734689)

[Volatility Switch ERPE Results 4](#_Toc50734690)

[Effect of Removing Participants with ADHD and Depression: 4](#_Toc50734691)

[Full Statistical Models: 5](#_Toc50734692)

[Accuracy Mixed Model 5](#_Toc50734693)

[Time Spent Moving Mixed Model 8](#_Toc50734694)

[Speed Mixed Model 10](#_Toc50734695)

[Acceleration Mixed Model 13](#_Toc50734696)

[Jerk Mixed Model 17](#_Toc50734697)

[Number of Turns Mixed Model 20](#_Toc50734698)

[Number of Dominant Policy Turns Mixed Model 22](#_Toc50734699)

[Number of Hypothesis Switches Mixed Model 25](#_Toc50734700)

[Average Prediction Error Mixed Model: 28](#_Toc50734701)

[Condition-wise Prediction Error Slope Mixed Model: Accuracy Not Included 31](#_Toc50734702)

[Condition-wise Prediction Error Mixed Model: Accuracy Random Effect Included 33](#_Toc50734703)

[Prediction Error Slope by Accuracy and Agency Mixed Model 36](#_Toc50734704)

[Volatility ERPE Mixed Model 42](#_Toc50734705)

[Hypothesis Switch ERPE Mixed Model 46](#_Toc50734706)

[Incorrect Trials Only Hypothesis Switch ERPE Mixed Model 57](#_Toc50734707)

[Incorrect Trials Only Hypothesis Switch ERPE Figure 62](#_Toc50734708)

# Policy Definitions:

## Horizontal


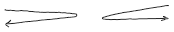


Any turn (surrounded on both sides by at least three frames of direction maintenance) in which the start and end directions are either east or west (right or left).

## Vertical


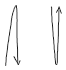


Any turn (surrounded on both sides by at least three frames of direction maintenance) in which the start and end directions are either east or west (right or left).

## Perpendicular-Cardinal


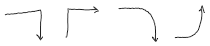


Any turn (surrounded on both sides by at least three frames of direction maintenance) in which the start and end directions are any of the four cardinal directions (right/ left/up/down).

*OR*

Three successive* turns, whose directions move clockwise or anticlockwise but is too short to count as a circle, where the maintenance around the first and last turns are in cardinal directions. This is a *rounded corner* starting and ending in cardinal directions. These get counted as one turn.

## Non-Cardinal


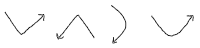


Any turn (surrounded on both sides by at least three frames of direction maintenance) in which the start and end directions are NOT any of the four cardinal directions (diagonals).

*OR*

Three successive* turns, whose directions move clockwise or anticlockwise but is too short to count as a circle, where the maintenance around the first and last turns are NOT in cardinal directions. This is a *rounded corner* starting and ending in diagonals. These get counted as one turn.

## Hesitant-Straight


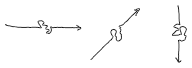


Any time when the direction of travel changes for a little bit, but the directions of the maintenance around the ‘hesitation’ are in the same direction. Maintenance in any direction in the middle of these cannot be longer than 3 frames (otherwise the turns get counted separately). Can be found in the middle of what is otherwise defined as a rounded turn or a circle also. This counts as a ‘turn’, because it indicates a decision to maintain direction. Likely occurs frequently as a result of picking up and moving the laser mouse while not intending to change direction at all.

## Circle


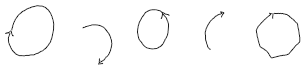


Four successive (not counting adjust-maintains in between) turns whose directions move clockwise or anticlockwise. This counts anything that is at least ¾ of a semi-circle in shape. Each full circle (or any qualifying part of a first circle) gets counted as one turn for this category. Circles are surrounded by at least 3 frames of maintenance in directions non-consistent with a circle, or in the opposite direction of rotation.

*where hesitant-straights in the middle get skipped for counting and labelling this

# Biased-nearest-object Method for Hypothesis Definition:

For each frame, the shortest Euclidean distance between the eyes and any square was taken to be the square at which the participant was looking, which was taken as a proxy for their *hypothesis* about which square they controlled at that point in time. When two squares were close together (distance to eye positions is less than one squares’ length different) and when the nearest square to each eye differed, the hypothesis was biased towards the square which was hypothesised immediately prior. If this was not within the scope of the nearest squares, the nearest square to the average location of the two eyes was chosen.

# Interaction Results for Movement and Strategy Variables:

Where interactions are not reported here, there were no significant interactions.

## Speed

There was a significant interaction between variability and volatility (F(1,4661)=16.36, p<0.001). Post-hoc analyses showed that in the low variability/high volatility condition, participants moved faster than the low variability/low volatility (z=3.89, p<0.001), the high variability/high volatility (z=7.18, p<0.001), and the high variability/low volatility conditions (z=5.31, p<0.001). Further, participants moved faster when both variability and volatility were low than when they were both high (z=3.22, p=0.008).

## Turn Count

There was a significant interaction between variability and volatility (F(1,4661)=4.47, p=0.034). Post-hoc analysis showed the effect of variability was stronger in low volatility conditions (all pairwise contrasts between conditions were significant, z>11.74, p<0.001, except low/low vs low/high and high/low vs. high/high which were non-significant).

## Hypothesis Switch Count

There was also a significant interaction between variability and volatility (**Figure 5**; F(1,4661)=6.45, p=0.011). Post-hoc tests showed that in low variability conditions only, participants switched hypotheses more often under high volatility than low volatility (z=2.79, p=0.031).

# Volatility Switch ERPE Results

To look at whether changes to the variability distribution due to volatility lead to any differences in prediction error, we performed an MLM on the ERPE centered on volatility switches. In addition to the standard MLM, we included the fixed effect of time-bin, and an additional fixed effect of average prediction error as a covariate. Of the fixed effects of interest, only variability was marginally significant (F(1,662) = 3.91, p=0.05), such that around the time of volatility switches, high variability trials had greater prediction error than low variability trials (t(662)=1.98, p=0.049). The lack of interaction with time-bin suggests this difference was not temporally sensitive to the onset of a new variability distribution and similar patterns across uncertainty conditions and autism traits.

# Effect of Removing Participants with ADHD and Depression:

All models were re-examined with these two participants removed. The following bullet-points report the fixed effects for which the significant effects were altered by removing these two participants.

- For the mixed model using the number of turns per trial as a dependent variable, removing these participants results in a new significant interaction between variability and AQ (F(1,4441)=4.58, p=0.032). This is down from insignificant p = 0.074 in reported sample. Simple effects showed that the difference in variability conditions decreased with increasing AQ score.
- The significant three-way interaction between variability, volatility and AQ for dominant policy use is lost (F(1,4441)=3.23, p=0.072). Up from p = 0.039 in reported sample.
- The marginally significant main effect of volatility for condition-wise prediction error slope is lost (F(1,108)=3.79, p=0.054).
- The marginally significant main effect of variability for the volatility centered ERPE is lost (F(1,630)=3.04, p=0.082).

# Full Statistical Models:

For variability and volatility coding, 0 is low, 1 is high.

## Accuracy Mixed Model

Mixed Model

| Model Info | | | |
| --- | --- | --- | --- |
|  |  |  |  |
| **Info** | |  | |
| Estimate |  | Linear mixed model fit by REML |  |
| Call |  | Accuracy ~ 1 + Variability + Volatility + AQ + Variability:Volatility + AQ:Volatility + AQ:Variability + AQ:Volatility:Variability+( 1 \| id ) |  |
| AIC |  | 4238.694 |  |
| R-squared Marginal |  | 0.019 |  |
| R-squared Conditional |  | 0.068 |  |
|  | | | |

Model Results

| Fixed Effect Omnibus tests | | | | | | | | | | | | | | | | | | | | | | | | | | | | |  |  |  |  |  |  |  |  |  |  |
| --- | --- | --- | --- | --- | --- | --- | --- | --- | --- | --- | --- | --- | --- | --- | --- | --- | --- | --- | --- | --- | --- | --- | --- | --- | --- | --- | --- | --- | --- | --- | --- | --- | --- | --- | --- | --- | --- | --- |
|  | | | | |  | | | |  | | | |  |  | | | |  | |  | | | | |  | |  |  |  |  |  |  |  |  |  |  |  |  |
|  | | | | | | | | | **F** | | | | | **Num df** | | | | | | **Den df** | | | | | | | **p** | |  |  |  |  |  |  |  |  |  |  |
| Variability | | | | |  | | | | 85.068 | | | |  | 1 | | | |  | | 4664.477 | | | | |  | | < .001 |  |  |  |  |  |  |  |  |  |  |  |
| Volatility | | | | |  | | | | 0.130 | | | |  | 1 | | | |  | | 4664.483 | | | | |  | | 0.718 |  |  |  |  |  |  |  |  |  |  |  |
| AQ | | | | |  | | | | 0.057 | | | |  | 1 | | | |  | | 37.215 | | | | |  | | 0.813 |  |  |  |  |  |  |  |  |  |  |  |
| Variability ✻ Volatility | | | | |  | | | | 8.617 | | | |  | 1 | | | |  | | 4664.578 | | | | |  | | 0.003 |  |  |  |  |  |  |  |  |  |  |  |
| Volatility ✻ AQ | | | | |  | | | | 0.041 | | | |  | 1 | | | |  | | 4662.239 | | | | |  | | 0.840 |  |  |  |  |  |  |  |  |  |  |  |
| Variability ✻ AQ | | | | |  | | | | 0.585 | | | |  | 1 | | | |  | | 4665.167 | | | | |  | | 0.445 |  |  |  |  |  |  |  |  |  |  |  |
| Variability ✻ Volatility ✻ AQ | | | | |  | | | | 0.682 | | | |  | 1 | | | |  | | 4664.549 | | | | |  | | 0.409 |  |  |  |  |  |  |  |  |  |  |  |
| Note. Satterthwaite method for degrees of freedom | | | | | | | | | | | | | | | | | | | | | | | | | | | | |  |  |  |  |  |  |  |  |  |  |
|  | | | | | | | | | | | | | | | | | | | | | | | | | | | | |  |  |  |  |  |  |  |  |  |  |
| Fixed Effects Parameter Estimates | | | | | | | | | | | | | | | | | | | | | | | | | | | | | | | | | | | |  |  |  |
|  | | | | | | | | | | | | | | | | | | | | | | | **95% Confidence Interval** | | | | | |  | | | | | | |  |  |  |
| **Names** | | | | **Effect** | | | | | | | **Estimate** | | | | | | **SE** | | | | | | **Lower** | | | **Upper** | | | **df** | | **t** | | | **p** | | | |  |
| (Intercept) | | |  | (Intercept) | | | |  | | | 0.817 | | | |  | | 0.015 | | | |  | | 0.789 |  | | 0.846 | |  | 37.382 |  | | 56.026 |  | | < .001 | |  | |
| Variability1 | | |  | 1 - 0 | | | |  | | | -0.101 | | | |  | | 0.011 | | | |  | | -0.122 |  | | -0.079 | |  | 4664.477 |  | | -9.223 |  | | < .001 | |  | |
| Volatility1 | | |  | 1 - 0 | | | |  | | | -0.004 | | | |  | | 0.011 | | | |  | | -0.025 |  | | 0.017 | |  | 4664.483 |  | | -0.361 |  | | 0.718 | |  | |
| AQ | | |  | AQ | | | |  | | | 5.958e-4 | | | |  | | 0.003 | | | |  | | -0.004 |  | | 0.006 | |  | 37.215 |  | | 0.238 |  | | 0.813 | |  | |
| Variability1 ✻ Volatility1 | | |  | 1 - 0 ✻ 1 - 0 | | | |  | | | -0.064 | | | |  | | 0.022 | | | |  | | -0.107 |  | | -0.021 | |  | 4664.578 |  | | -2.935 |  | | 0.003 | |  | |
| Volatility1 ✻ AQ | | |  | 1 - 0 ✻ AQ | | | |  | | | -3.758e−4 | | | |  | | 0.002 | | | |  | | -0.004 |  | | 0.003 | |  | 4662.239 |  | | -0.202 |  | | 0.840 | |  | |
| Variability1 ✻ AQ | | |  | 1 - 0 ✻ AQ | | | |  | | | 0.001 | | | |  | | 0.002 | | | |  | | -0.002 |  | | 0.005 | |  | 4665.167 |  | | 0.765 |  | | 0.445 | |  | |
| Variability1 ✻ Volatility1 ✻ AQ | | |  | 1 - 0 ✻ 1 - 0 ✻ AQ | | | |  | | | -0.003 | | | |  | | 0.004 | | | |  | | -0.010 |  | | 0.004 | |  | 4664.549 |  | | -0.826 |  | | 0.409 | |  | |
|  | | | | | | | | | | | | | | | | | | | | | | | | | | | | | | | | | | | |  |  |  |
| Random Components | | | | | | | | | | | | | | | | | | | | | |  |  |  |  |  |  |  |  |  |  |  |  |  |  |  |  |  |
|  |  |  | | | |  |  | | |  | |  | | |  |  | | |  | | |  |  |  |  |  |  |  |  |  |  |  |  |  |  |  |  |  |
| **Groups** | | **Name** | | | | | **SD** | | | | | **Variance** | | | | **ICC** | | | | | |  |  |  |  |  |  |  |  |  |  |  |  |  |  |  |  |  |
| id |  | (Intercept) | | | |  | 0.085 | | |  | | 0.007 | | |  | 0.050 | | |  | | |  |  |  |  |  |  |  |  |  |  |  |  |  |  |  |  |  |
| Residual |  |  | | | |  | 0.374 | | |  | | 0.140 | | |  |  | | |  | | |  |  |  |  |  |  |  |  |  |  |  |  |  |  |  |  |  |
| Note. Number of Obs: 4707 , groups: id , 40 | | | | | | | | | | | | | | | | | | | | | |  |  |  |  |  |  |  |  |  |  |  |  |  |  |  |  |  |
|  | | | | | | | | | | | | | | | | | | | | | |  |  |  |  |  |  |  |  |  |  |  |  |  |  |  |  |  |

Post Hoc Tests

| Post Hoc Comparisons - Variability ✻ Volatility | | | | | | | | | | | | | | | | | |
| --- | --- | --- | --- | --- | --- | --- | --- | --- | --- | --- | --- | --- | --- | --- | --- | --- | --- |
| **Comparison** | | | | | | | | | |  | | | | | | | |
| **Variability** | | **Volatility** | |  | | **Variability** | | **Volatility** | | **Difference** | | **SE** | | **z** | | **p_bonferroni_** | |
| 0 |  | 0 |  | - |  | 0 |  | 1 |  | -0.028 |  | 0.016 |  | -1.811 |  | 0.421 |  |
| 0 |  | 0 |  | - |  | 1 |  | 0 |  | 0.069 |  | 0.016 |  | 4.414 |  | < .001 |  |
| 0 |  | 0 |  | - |  | 1 |  | 1 |  | 0.105 |  | 0.015 |  | 6.785 |  | < .001 |  |
| 0 |  | 1 |  | - |  | 1 |  | 1 |  | 0.133 |  | 0.015 |  | 8.661 |  | < .001 |  |
| 1 |  | 0 |  | - |  | 0 |  | 1 |  | -0.097 |  | 0.015 |  | -6.260 |  | < .001 |  |
| 1 |  | 0 |  | - |  | 1 |  | 1 |  | 0.036 |  | 0.015 |  | 2.343 |  | 0.115 |  |
|  | | | | | | | | | | | | | | | | | |

## Time Spent Moving Mixed Model

Mixed Model

| Model Info | | | |
| --- | --- | --- | --- |
|  |  |  |  |
| **Info** | |  | |
| Estimate |  | Linear mixed model fit by REML |  |
| Call |  | TimeSpentMoving ~ 1 + AQ + Volatility + Variability + TimetoMovement + Volatility:Variability + AQ:Variability + AQ:Volatility + AQ:Variability:Volatility+( 1 \| id ) |  |
| AIC |  | 13750.596 |  |
| R-squared Marginal |  | 0.127 |  |
| R-squared Conditional |  | 0.559 |  |
|  | | | |

Model Results

| Fixed Effect Omnibus tests | | | | | | | | | |
| --- | --- | --- | --- | --- | --- | --- | --- | --- | --- |
|  |  |  |  |  |  |  |  |  |  |
|  | | **F** | | **Num df** | | **Den df** | | **p** | |
| AQ |  | 0.374 |  | 1 |  | 38.003 |  | 0.544 |  |
| Volatility |  | 0.251 |  | 1 |  | 4660.287 |  | 0.616 |  |
| Variability |  | 727.707 |  | 1 |  | 4660.277 |  | < .001 |  |
| TimetoMovement |  | 431.902 |  | 1 |  | 4674.855 |  | < .001 |  |
| Volatility ✻ Variability |  | 2.694 |  | 1 |  | 4660.280 |  | 0.101 |  |
| AQ ✻ Variability |  | 2.489 |  | 1 |  | 4660.325 |  | 0.115 |  |
| AQ ✻ Volatility |  | 1.702 |  | 1 |  | 4660.132 |  | 0.192 |  |
| AQ ✻ Volatility ✻ Variability |  | 11.367 |  | 1 |  | 4660.309 |  | < .001 |  |
| Note. Satterthwaite method for degrees of freedom | | | | | | | | | |
|  | | | | | | | | | |

| Fixed Effects Parameter Estimates | | | | | | | | | | | | | | | | | |
| --- | --- | --- | --- | --- | --- | --- | --- | --- | --- | --- | --- | --- | --- | --- | --- | --- | --- |
|  | | | | | | | | **95% Confidence Interval** | | | |  | | | | | |
| **Names** | | **Effect** | | **Estimate** | | **SE** | | **Lower** | | **Upper** | | **df** | | **t** | | **p** | |
| (Intercept) |  | (Intercept) |  | 13.721 |  | 0.160 |  | 13.408 |  | 14.035 |  | 38.013 |  | 85.780 |  | < .001 |  |
| AQ |  | AQ |  | -0.017 |  | 0.027 |  | -0.071 |  | 0.037 |  | 38.003 |  | -0.612 |  | 0.544 |  |
| Volatility1 |  | 1 - 0 |  | -0.015 |  | 0.030 |  | -0.073 |  | 0.043 |  | 4660.287 |  | -0.501 |  | 0.616 |  |
| Variability1 |  | 1 - 0 |  | 0.801 |  | 0.030 |  | 0.743 |  | 0.860 |  | 4660.277 |  | 26.976 |  | < .001 |  |
| TimetoMovement |  | TimetoMovement |  | -0.940 |  | 0.045 |  | -1.029 |  | -0.852 |  | 4674.855 |  | -20.782 |  | < .001 |  |
| Volatility1 ✻ Variability1 |  | 1 - 0 ✻ 1 - 0 |  | -0.098 |  | 0.059 |  | -0.214 |  | 0.019 |  | 4660.280 |  | -1.641 |  | 0.101 |  |
| AQ ✻ Variability1 |  | AQ ✻ 1 - 0 |  | 0.008 |  | 0.005 |  | -0.002 |  | 0.018 |  | 4660.325 |  | 1.578 |  | 0.115 |  |
| AQ ✻ Volatility1 |  | AQ ✻ 1 - 0 |  | -0.007 |  | 0.005 |  | -0.017 |  | 0.003 |  | 4660.132 |  | -1.305 |  | 0.192 |  |
| AQ ✻ Volatility1 ✻ Variability1 |  | AQ ✻ 1 - 0 ✻ 1 - 0 |  | -0.034 |  | 0.010 |  | -0.054 |  | -0.014 |  | 4660.309 |  | -3.371 |  | < .001 |  |
|  | | | | | | | | | | | | | | | | | |

| Random Components | | | | | | | | | |
| --- | --- | --- | --- | --- | --- | --- | --- | --- | --- |
|  |  |  |  |  |  |  |  |  |  |
| **Groups** | | **Name** | | **SD** | | **Variance** | | **ICC** | |
| id |  | (Intercept) |  | 1.007 |  | 1.014 |  | 0.495 |  |
| Residual |  |  |  | 1.017 |  | 1.035 |  |  |  |
| Note. Number of Obs: 4707 , groups: id , 40 | | | | | | | | | |
|  | | | | | | | | | |

Simple Effects

| Simple effects of Volatility : Omnibus Tests | | | | | | | | | |
| --- | --- | --- | --- | --- | --- | --- | --- | --- | --- |
| **Moderator levels** | | | |  | | | | | |
| **Variability** | | **AQ** | | **X²** | | **df** | | **p** | |
| 0 |  | Mean-1·SD |  | 0.215 |  | 1.000 |  | 0.643 |  |
|  |  | Mean |  | 0.643 |  | 1.000 |  | 0.423 |  |
|  |  | Mean+1·SD |  | 2.594 |  | 1.000 |  | 0.107 |  |
| 1 |  | Mean-1·SD |  | 1.617 |  | 1.000 |  | 0.204 |  |
|  |  | Mean |  | 2.319 |  | 1.000 |  | 0.128 |  |
|  |  | Mean+1·SD |  | 11.553 |  | 1.000 |  | < .001 |  |
|  | | | | | | | | | |

| Simple effects of Volatility : Parameter estimates | | | | | | | | | | | | | | | | | |
| --- | --- | --- | --- | --- | --- | --- | --- | --- | --- | --- | --- | --- | --- | --- | --- | --- | --- |
| **Moderator levels** | | | |  | | | | | | **95% Confidence Interval** | | | |  | | | |
| **Variability** | | **AQ** | | **contrast** | | **Estimate** | | **SE** | | **Lower** | | **Upper** | | **z** | | **p** | |
| 0 |  | Mean-1·SD |  | 1 - 0 |  | -0.028 |  | 0.060 |  | -0.145 |  | 0.089 |  | -0.463 |  | 0.643 |  |
|  |  | Mean |  | 1 - 0 |  | 0.034 |  | 0.042 |  | -0.049 |  | 0.117 |  | 0.802 |  | 0.423 |  |
|  |  | Mean+1·SD |  | 1 - 0 |  | 0.095 |  | 0.059 |  | -0.021 |  | 0.211 |  | 1.611 |  | 0.107 |  |
| 1 |  | Mean-1·SD |  | 1 - 0 |  | 0.075 |  | 0.059 |  | -0.041 |  | 0.191 |  | 1.271 |  | 0.204 |  |
|  |  | Mean |  | 1 - 0 |  | -0.064 |  | 0.042 |  | -0.146 |  | 0.018 |  | -1.523 |  | 0.128 |  |
|  |  | Mean+1·SD |  | 1 - 0 |  | -0.203 |  | 0.060 |  | -0.319 |  | -0.086 |  | -3.399 |  | < .001 |  |
| Note. Simple effects are estimated keeping constant other independent variable(s) in the model | | | | | | | | | | | | | | | | | |
|  | | | | | | | | | | | | | | | | | |

## Speed Mixed Model

Mixed Model

| Model Info | | | |
| --- | --- | --- | --- |
|  |  |  |  |
| **Info** | |  | |
| Estimate |  | Linear mixed model fit by REML |  |
| Call |  | MeanSpeed ~ 1 + Variability + Volatility + AQ + Variability:Volatility + Variability:AQ + Volatility:AQ + Variability:Volatility:AQ+( 1 \| id ) |  |
| AIC |  | 26650.527 |  |
| R-squared Marginal |  | 0.041 |  |
| R-squared Conditional |  | 0.641 |  |
|  | | | |

Model Results

| Fixed Effect Omnibus tests | | | | | | | | | |
| --- | --- | --- | --- | --- | --- | --- | --- | --- | --- |
|  |  |  |  |  |  |  |  |  |  |
|  | | **F** | | **Num df** | | **Den df** | | **p** | |
| Variability |  | 36.418 |  | 1 |  | 4661.161 |  | < .001 |  |
| Volatility |  | 2.196 |  | 1 |  | 4661.164 |  | 0.138 |  |
| AQ |  | 2.443 |  | 1 |  | 38.004 |  | 0.126 |  |
| Variability ✻ Volatility |  | 16.359 |  | 1 |  | 4661.166 |  | < .001 |  |
| Variability ✻ AQ |  | 0.823 |  | 1 |  | 4661.186 |  | 0.364 |  |
| Volatility ✻ AQ |  | 0.082 |  | 1 |  | 4661.079 |  | 0.775 |  |
| Variability ✻ Volatility ✻ AQ |  | 0.687 |  | 1 |  | 4661.165 |  | 0.407 |  |
| Note. Satterthwaite method for degrees of freedom | | | | | | | | | |
|  | | | | | | | | | |

| Fixed Effects Parameter Estimates | | | | | | | | | | | | | | | | | |
| --- | --- | --- | --- | --- | --- | --- | --- | --- | --- | --- | --- | --- | --- | --- | --- | --- | --- |
|  | | | | | | | | **95% Confidence Interval** | | | |  | | | | | |
| **Names** | | **Effect** | | **Estimate** | | **SE** | | **Lower** | | **Upper** | | **df** | | **t** | | **p** | |
| (Intercept) |  | (Intercept) |  | 13.115 |  | 0.820 |  | 11.508 |  | 14.723 |  | 38.011 |  | 15.991 |  | < .001 |  |
| Variability1 |  | 1 - 0 |  | -0.706 |  | 0.117 |  | -0.935 |  | -0.477 |  | 4661.161 |  | -6.035 |  | < .001 |  |
| Volatility1 |  | 1 - 0 |  | 0.173 |  | 0.117 |  | -0.056 |  | 0.403 |  | 4661.164 |  | 1.482 |  | 0.138 |  |
| AQ |  | AQ |  | -0.220 |  | 0.141 |  | -0.497 |  | 0.056 |  | 38.004 |  | -1.563 |  | 0.126 |  |
| Variability1 ✻ Volatility1 |  | 1 - 0 ✻ 1 - 0 |  | -0.947 |  | 0.234 |  | -1.405 |  | -0.488 |  | 4661.166 |  | -4.045 |  | < .001 |  |
| Variability1 ✻ AQ |  | 1 - 0 ✻ AQ |  | -0.018 |  | 0.020 |  | -0.057 |  | 0.021 |  | 4661.186 |  | -0.907 |  | 0.364 |  |
| Volatility1 ✻ AQ |  | 1 - 0 ✻ AQ |  | -0.006 |  | 0.020 |  | -0.045 |  | 0.033 |  | 4661.079 |  | -0.286 |  | 0.775 |  |
| Variability1 ✻ Volatility1 ✻ AQ |  | 1 - 0 ✻ 1 - 0 ✻ AQ |  | 0.033 |  | 0.040 |  | -0.045 |  | 0.111 |  | 4661.165 |  | 0.829 |  | 0.407 |  |
|  | | | | | | | | | | | | | | | | | |

| Random Components | | | | | | | | | |
| --- | --- | --- | --- | --- | --- | --- | --- | --- | --- |
|  |  |  |  |  |  |  |  |  |  |
| **Groups** | | **Name** | | **SD** | | **Variance** | | **ICC** | |
| id |  | (Intercept) |  | 5.173 |  | 26.758 |  | 0.625 |  |
| Residual |  |  |  | 4.007 |  | 16.054 |  |  |  |
| Note. Number of Obs: 4707 , groups: id , 40 | | | | | | | | | |
|  | | | | | | | | | |

Post Hoc Tests

| Post Hoc Comparisons - Variability ✻ Volatility | | | | | | | | | | | | | | | | | |
| --- | --- | --- | --- | --- | --- | --- | --- | --- | --- | --- | --- | --- | --- | --- | --- | --- | --- |
| **Comparison** | | | | | | | | | |  | | | | | | | |
| **Variability** | | **Volatility** | |  | | **Variability** | | **Volatility** | | **Difference** | | **SE** | | **z** | | **p_bonferroni_** | |
| 0 |  | 0 |  | - |  | 0 |  | 1 |  | -0.647 |  | 0.166 |  | -3.888 |  | < .001 |  |
| 0 |  | 0 |  | - |  | 1 |  | 0 |  | 0.233 |  | 0.167 |  | 1.397 |  | 0.975 |  |
| 0 |  | 0 |  | - |  | 1 |  | 1 |  | 0.533 |  | 0.165 |  | 3.223 |  | 0.008 |  |
| 0 |  | 1 |  | - |  | 1 |  | 1 |  | 1.179 |  | 0.164 |  | 7.180 |  | < .001 |  |
| 1 |  | 0 |  | - |  | 0 |  | 1 |  | -0.879 |  | 0.166 |  | -5.309 |  | < .001 |  |
| 1 |  | 0 |  | - |  | 1 |  | 1 |  | 0.300 |  | 0.165 |  | 1.822 |  | 0.411 |  |
|  | | | | | | | | | | | | | | | | | |

| Post Hoc Comparisons - Variability | | | | | | | | | | | | | |
| --- | --- | --- | --- | --- | --- | --- | --- | --- | --- | --- | --- | --- | --- |
| **Comparison** | | | | | |  | | | | | | | |
| **Variability** | |  | | **Variability** | | **Difference** | | **SE** | | **z** | | **p_bonferroni_** | |
| 0 |  | - |  | 1 |  | 0.706 |  | 0.117 |  | 6.035 |  | < .001 |  |
|  | | | | | | | | | | | | | |

## Acceleration Mixed Model

Mixed Model

| Model Info | | | |
| --- | --- | --- | --- |
|  |  |  |  |
| **Info** | |  | |
| Estimate |  | Linear mixed model fit by REML |  |
| Call |  | Acceleration ~ 1 + Variability + Volatility + AQ + Variability:Volatility + Variability:AQ + Volatility:AQ + Variability:Volatility:AQ+( 1 \| id ) |  |
| AIC |  | -23077.864 |  |
| R-squared Marginal |  | 0.013 |  |
| R-squared Conditional |  | 0.077 |  |
|  | | | |

Model Results

| Fixed Effect Omnibus tests | | | | | | | | | |
| --- | --- | --- | --- | --- | --- | --- | --- | --- | --- |
|  |  |  |  |  |  |  |  |  |  |
|  | | **F** | | **Num df** | | **Den df** | | **p** | |
| Variability |  | 12.677 |  | 1 |  | 4664.138 |  | < .001 |  |
| Volatility |  | 0.779 |  | 1 |  | 4664.152 |  | 0.377 |  |
| AQ |  | 5.729 |  | 1 |  | 37.799 |  | 0.022 |  |
| Variability ✻ Volatility |  | 1.056 |  | 1 |  | 4664.221 |  | 0.304 |  |
| Variability ✻ AQ |  | 0.007 |  | 1 |  | 4664.674 |  | 0.932 |  |
| Volatility ✻ AQ |  | 0.379 |  | 1 |  | 4662.388 |  | 0.538 |  |
| Variability ✻ Volatility ✻ AQ |  | 0.247 |  | 1 |  | 4664.197 |  | 0.619 |  |
| Note. Satterthwaite method for degrees of freedom | | | | | | | | | |
|  | | | | | | | | | |

| Fixed Effects Parameter Estimates | | | | | | | | | | | | | | | | | |
| --- | --- | --- | --- | --- | --- | --- | --- | --- | --- | --- | --- | --- | --- | --- | --- | --- | --- |
|  | | | | | | | | **95% Confidence Interval** | | | |  | | | | | |
| **Names** | | **Effect** | | **Estimate** | | **SE** | | **Lower** | | **Upper** | | **df** | | **t** | | **p** | |
| (Intercept) |  | (Intercept) |  | 0.013 |  | 8.981e-4 |  | 0.011 |  | 0.014 |  | 37.935 |  | 14.088 |  | < .001 |  |
| Variability1 |  | 1 - 0 |  | 0.002 |  | 5.964e-4 |  | 9.545e-4 |  | 0.003 |  | 4664.138 |  | 3.560 |  | < .001 |  |
| Volatility1 |  | 1 - 0 |  | -5.265e−4 |  | 5.963e-4 |  | -0.002 |  | 6.423e-4 |  | 4664.152 |  | -0.883 |  | 0.377 |  |
| AQ |  | AQ |  | -3.691e−4 |  | 1.542e-4 |  | -6.713e−4 |  | -6.684e−5 |  | 37.799 |  | -2.393 |  | 0.022 |  |
| Variability1 ✻ Volatility1 |  | 1 - 0 ✻ 1 - 0 |  | -0.001 |  | 0.001 |  | -0.004 |  | 0.001 |  | 4664.221 |  | -1.028 |  | 0.304 |  |
| Variability1 ✻ AQ |  | 1 - 0 ✻ AQ |  | 8.737e-6 |  | 1.018e-4 |  | -1.908e−4 |  | 2.083e-4 |  | 4664.674 |  | 0.086 |  | 0.932 |  |
| Volatility1 ✻ AQ |  | 1 - 0 ✻ AQ |  | 6.260e-5 |  | 1.017e-4 |  | -1.368e−4 |  | 2.620e-4 |  | 4662.388 |  | 0.615 |  | 0.538 |  |
| Variability1 ✻ Volatility1 ✻ AQ |  | 1 - 0 ✻ 1 - 0 ✻ AQ |  | -1.012e−4 |  | 2.036e-4 |  | -5.003e−4 |  | 2.978e-4 |  | 4664.197 |  | -0.497 |  | 0.619 |  |
|  | | | | | | | | | | | | | | | | | |

| Random Components | | | | | | | | | |
| --- | --- | --- | --- | --- | --- | --- | --- | --- | --- |
|  |  |  |  |  |  |  |  |  |  |
| **Groups** | | **Name** | | **SD** | | **Variance** | | **ICC** | |
| id |  | (Intercept) |  | 0.005 |  | 2.867e-5 |  | 0.064 |  |
| Residual |  |  |  | 0.020 |  | 4.172e-4 |  |  |  |
| Note. Number of Obs: 4707 , groups: id , 40 | | | | | | | | | |
|  | | | | | | | | | |

Post Hoc Tests

| Post Hoc Comparisons - Variability | | | | | | | | | | | | | |
| --- | --- | --- | --- | --- | --- | --- | --- | --- | --- | --- | --- | --- | --- |
| **Comparison** | | | | | |  | | | | | | | |
| **Variability** | |  | | **Variability** | | **Difference** | | **SE** | | **z** | | **p_bonferroni_** | |
| 0 |  | - |  | 1 |  | -0.002 |  | 5.964e-4 |  | -3.560 |  | < .001 |  |
|  | | | | | | | | | | | | | |

Post Hoc Tests

| Post Hoc Comparisons - Variability | | | | | | | | | | | | | |
| --- | --- | --- | --- | --- | --- | --- | --- | --- | --- | --- | --- | --- | --- |
| **Comparison** | | | | | |  | | | | | | | |
| **Variability** | |  | | **Variability** | | **Difference** | | **SE** | | **z** | | **p_bonferroni_** | |
| 0 |  | - |  | 1 |  | -0.0021233 |  | 5.9637e-4 |  | -3.5604 |  | < .001 |  |
|  | | | | | | | | | | | | | |

 Linear Regression

| Model Fit Measures | | | | | |
| --- | --- | --- | --- | --- | --- |
|  |  |  |  |  |  |
| **Model** | | **R** | | **R²** | |
| 1 |  | 0.104 |  | 0.011 |  |
|  | | | | | |

| Model Coefficients - Acceleration | | | | | | | | | |
| --- | --- | --- | --- | --- | --- | --- | --- | --- | --- |
|  |  |  |  |  |  |  |  |  |  |
| **Predictor** | | **Estimate** | | **SE** | | **t** | | **p** | |
| Intercept |  | 0.021 |  | 0.001 |  | 17.713 |  | < .001 |  |
| AQ |  | -3.767e−4 |  | 5.248e-5 |  | -7.178 |  | < .001 |  |
|  | | | | | | | | | |

## Jerk Mixed Model

| Model Info | | | |
| --- | --- | --- | --- |
|  |  |  |  |
| **Info** | |  | |
| Estimate |  | Linear mixed model fit by REML |  |
| Call |  | Jerk ~ 1 + Variability + Volatility + AQ + Variability:Volatility + Variability:AQ + Volatility:AQ + Variability:Volatility:AQ+( 1 \| id ) |  |
| AIC |  | -32582 |  |
| R-squared Marginal |  | 8.5604e-4 |  |
| R-squared Conditional |  | 8.5604e-4 |  |
| Note. Results may be uninterpretable or misleading. Try to refine your model. | | | |
| Note. singular fit | | | |
|  | | | |

Model Results

| Fixed Effect Omnibus tests | | | | | | | | | |
| --- | --- | --- | --- | --- | --- | --- | --- | --- | --- |
|  |  |  |  |  |  |  |  |  |  |
|  | | **F** | | **Num df** | | **Den df** | | **p** | |
| Variability |  | 0.257546 |  | 1 |  | 4699.0 |  | 0.612 |  |
| Volatility |  | 0.122248 |  | 1 |  | 4699.0 |  | 0.727 |  |
| AQ |  | 0.282417 |  | 1 |  | 4699.0 |  | 0.595 |  |
| Variability ✻ Volatility |  | 0.987322 |  | 1 |  | 4699.0 |  | 0.320 |  |
| Variability ✻ AQ |  | 1.483438 |  | 1 |  | 4699.0 |  | 0.223 |  |
| Volatility ✻ AQ |  | 0.024998 |  | 1 |  | 4699.0 |  | 0.874 |  |
| Variability ✻ Volatility ✻ AQ |  | 0.869415 |  | 1 |  | 4699.0 |  | 0.351 |  |
| Note. singular fit | | | | | | | | | |
| Note. Satterthwaite method for degrees of freedom | | | | | | | | | |
|  | | | | | | | | | |

| Fixed Effects Parameter Estimates | | | | | | | | | | | | | | | | | |
| --- | --- | --- | --- | --- | --- | --- | --- | --- | --- | --- | --- | --- | --- | --- | --- | --- | --- |
|  | | | | | | | | **95% Confidence Interval** | | | |  | | | | | |
| **Names** | | **Effect** | | **Estimate** | | **SE** | | **Lower** | | **Upper** | | **df** | | **t** | | **p** | |
| (Intercept) |  | (Intercept) |  | 1.3243e-4 |  | 1.0928e-4 |  | -8.1763e−5 |  | 3.4662e-4 |  | 4699.0 |  | 1.21179 |  | 0.226 |  |
| Variability1 |  | 1 - 0 |  | 1.1092e-4 |  | 2.1856e-4 |  | -3.1746e−4 |  | 5.3930e-4 |  | 4699.0 |  | 0.50749 |  | 0.612 |  |
| Volatility1 |  | 1 - 0 |  | 7.6419e-5 |  | 2.1856e-4 |  | -3.5196e−4 |  | 5.0480e-4 |  | 4699.0 |  | 0.34964 |  | 0.727 |  |
| AQ |  | AQ |  | 9.9132e-6 |  | 1.8654e-5 |  | -2.6648e−5 |  | 4.6474e-5 |  | 4699.0 |  | 0.53143 |  | 0.595 |  |
| Variability1 ✻ Volatility1 |  | 1 - 0 ✻ 1 - 0 |  | 4.3435e-4 |  | 4.3713e-4 |  | -4.2241e−4 |  | 0.0012911 |  | 4699.0 |  | 0.99364 |  | 0.320 |  |
| Variability1 ✻ AQ |  | 1 - 0 ✻ AQ |  | 4.5439e-5 |  | 3.7308e-5 |  | -2.7682e−5 |  | 1.1856e-4 |  | 4699.0 |  | 1.21796 |  | 0.223 |  |
| Volatility1 ✻ AQ |  | 1 - 0 ✻ AQ |  | 5.8986e-6 |  | 3.7308e-5 |  | -6.7223e−5 |  | 7.9020e-5 |  | 4699.0 |  | 0.15811 |  | 0.874 |  |
| Variability1 ✻ Volatility1 ✻ AQ |  | 1 - 0 ✻ 1 - 0 ✻ AQ |  | -6.9573e−5 |  | 7.4615e-5 |  | -2.1582e−4 |  | 7.6670e-5 |  | 4699.0 |  | -0.93242 |  | 0.351 |  |
|  | | | | | | | | | | | | | | | | | |

| Random Components | | | | | | | | | |
| --- | --- | --- | --- | --- | --- | --- | --- | --- | --- |
|  |  |  |  |  |  |  |  |  |  |
| **Groups** | | **Name** | | **SD** | | **Variance** | | **ICC** | |
| id |  | (Intercept) |  | 0.0000000 |  | 0.0000 |  | 0.0000 |  |
| Residual |  |  |  | 0.0074961 |  | 5.6191e-5 |  |  |  |
| Note. Number of Obs: 4707 , groups: id , 40 | | | | | | | | | |
|  | | | | | | | | | |

## Number of Turns Mixed Model

| Model Info | | | |
| --- | --- | --- | --- |
|  |  |  |  |
| **Info** | |  | |
| Estimate |  | Linear mixed model fit by REML |  |
| Call |  | nTurns ~ 1 + Variability + Volatility + AQ + Variability:Volatility + Variability:AQ + Volatility:AQ + Variability:Volatility:AQ+( 1 \| id ) |  |
| AIC |  | 35054.526405 |  |
| R-squared Marginal |  | 0.036379 |  |
| R-squared Conditional |  | 0.532821 |  |
|  | | | |

Model Results

| Fixed Effect Omnibus tests | | | | | | | | | |
| --- | --- | --- | --- | --- | --- | --- | --- | --- | --- |
|  |  |  |  |  |  |  |  |  |  |
|  | | **F** | | **Num df** | | **Den df** | | **p** | |
| Variability |  | 346.2197567 |  | 1 |  | 4661.184 |  | < .001 |  |
| Volatility |  | 0.0086040 |  | 1 |  | 4661.188 |  | 0.926 |  |
| AQ |  | 0.0675365 |  | 1 |  | 37.939 |  | 0.796 |  |
| Variability ✻ Volatility |  | 4.4744804 |  | 1 |  | 4661.192 |  | 0.034 |  |
| Variability ✻ AQ |  | 3.1868060 |  | 1 |  | 4661.224 |  | 0.074 |  |
| Volatility ✻ AQ |  | 0.5633213 |  | 1 |  | 4661.055 |  | 0.453 |  |
| Variability ✻ Volatility ✻ AQ |  | 3.7371077 |  | 1 |  | 4661.190 |  | 0.053 |  |
|  | | | | | | | | | |

| Fixed Effects Parameter Estimates | | | | | | | | | | | | | | | | | |
| --- | --- | --- | --- | --- | --- | --- | --- | --- | --- | --- | --- | --- | --- | --- | --- | --- | --- |
|  | | | | | | | | **95% Confidence Interval** | | | |  | | | | | |
| **Names** | | **Effect** | | **Estimate** | | **SE** | | **Lower** | | **Upper** | | **df** | | **t** | | **p** | |
| (Intercept) |  | (Intercept) |  | 35.435365 |  | 1.606677 |  | 32.2863359 |  | 38.5843951 |  | 37.949 |  | 22.055061 |  | < .001 |  |
| Variability1 |  | 1 - 0 |  | -5.333633 |  | 0.286647 |  | -5.8954505 |  | -4.7718154 |  | 4661.184 |  | -18.606981 |  | < .001 |  |
| Volatility1 |  | 1 - 0 |  | -0.026587 |  | 0.286632 |  | -0.5883752 |  | 0.5352006 |  | 4661.188 |  | -0.092758 |  | 0.926 |  |
| AQ |  | AQ |  | -0.071753 |  | 0.276104 |  | -0.6129075 |  | 0.4694008 |  | 37.939 |  | -0.259878 |  | 0.796 |  |
| Variability1 ✻ Volatility1 |  | 1 - 0 ✻ 1 - 0 |  | 1.212699 |  | 0.573300 |  | 0.0890523 |  | 2.3363465 |  | 4661.192 |  | 2.115297 |  | 0.034 |  |
| Variability1 ✻ AQ |  | 1 - 0 ✻ AQ |  | 0.087372 |  | 0.048944 |  | -0.0085554 |  | 0.1832997 |  | 4661.224 |  | 1.785163 |  | 0.074 |  |
| Volatility1 ✻ AQ |  | 1 - 0 ✻ AQ |  | 0.036691 |  | 0.048886 |  | -0.0591237 |  | 0.1325065 |  | 4661.055 |  | 0.750547 |  | 0.453 |  |
| Variability1 ✻ Volatility1 ✻ AQ |  | 1 - 0 ✻ 1 - 0 ✻ AQ |  | -0.189177 |  | 0.097859 |  | -0.3809762 |  | 0.0026230 |  | 4661.190 |  | -1.933160 |  | 0.053 |  |
|  | | | | | | | | | | | | | | | | | |

| Random Components | | | | | | | | | |
| --- | --- | --- | --- | --- | --- | --- | --- | --- | --- |
|  |  |  |  |  |  |  |  |  |  |
| **Groups** | | **Name** | | **SD** | | **Variance** | | **ICC** | |
| id |  | (Intercept) |  | 10.1187 |  | 102.388 |  | 0.51518 |  |
| Residual |  |  |  | 9.8160 |  | 96.353 |  |  |  |
|  | | | | | | | | | |

 Post Hoc Tests

| Post Hoc Comparisons - Variability ✻ Volatility | | | | | | | | | | | | | | | | | |
| --- | --- | --- | --- | --- | --- | --- | --- | --- | --- | --- | --- | --- | --- | --- | --- | --- | --- |
| **Comparison** | | | | | | | | | |  | | | | | | | |
| **Variability** | | **Volatility** | |  | | **Variability** | | **Volatility** | | **Difference** | | **SE** | | **z** | | **p_bonferroni_** | |
| 0 |  | 0 |  | - |  | 0 |  | 1 |  | 0.63294 |  | 0.40745 |  | 1.5534 |  | 0.722 |  |
| 0 |  | 0 |  | - |  | 1 |  | 0 |  | 5.93998 |  | 0.40833 |  | 14.5471 |  | < .001 |  |
| 0 |  | 0 |  | - |  | 1 |  | 1 |  | 5.36022 |  | 0.40492 |  | 13.2377 |  | < .001 |  |
| 0 |  | 1 |  | - |  | 1 |  | 1 |  | 4.72728 |  | 0.40241 |  | 11.7473 |  | < .001 |  |
| 1 |  | 0 |  | - |  | 0 |  | 1 |  | -5.30705 |  | 0.40582 |  | -13.0774 |  | < .001 |  |
| 1 |  | 0 |  | - |  | 1 |  | 1 |  | -0.57976 |  | 0.40328 |  | -1.4376 |  | 0.903 |  |
|  | | | | | | | | | | | | | | | | | |

| Post Hoc Comparisons - Variability | | | | | | | | | | | | | |
| --- | --- | --- | --- | --- | --- | --- | --- | --- | --- | --- | --- | --- | --- |
| **Comparison** | | | | | |  | | | | | | | |
| **Variability** | |  | | **Variability** | | **Difference** | | **SE** | | **z** | | **p_bonferroni_** | |
| 0 |  | - |  | 1 |  | 5.3336 |  | 0.28665 |  | 18.607 |  | < .001 |  |
|  | | | | | | | | | | | | | |

## Number of Dominant Policy Turns Mixed Model

Mixed Model

| Model Info | | | |
| --- | --- | --- | --- |
|  |  |  |  |
| **Info** | |  | |
| Estimate |  | Linear mixed model fit by REML |  |
| Call |  | nDomPol ~ 1 + nTurns + AQ + Variability + Volatility + Variability:Volatility + Variability:AQ + Volatility:AQ + Variability:Volatility:AQ+( 1 \| id ) |  |
| AIC |  | 29580.463 |  |
| R-squared Marginal |  | 0.576 |  |
| R-squared Conditional |  | 0.633 |  |
|  | | | |

Model Results

| Fixed Effect Omnibus tests | | | | | | | | | |
| --- | --- | --- | --- | --- | --- | --- | --- | --- | --- |
|  |  |  |  |  |  |  |  |  |  |
|  | | **F** | | **Num df** | | **Den df** | | **p** | |
| nTurns |  | 3839.669 |  | 1 |  | 3842.454 |  | < .001 |  |
| AQ |  | 0.039 |  | 1 |  | 36.935 |  | 0.845 |  |
| Variability |  | 0.704 |  | 1 |  | 4684.260 |  | 0.401 |  |
| Volatility |  | 1.807 |  | 1 |  | 4660.627 |  | 0.179 |  |
| Variability ✻ Volatility |  | 0.574 |  | 1 |  | 4660.975 |  | 0.449 |  |
| AQ ✻ Variability |  | 1.853 |  | 1 |  | 4661.195 |  | 0.174 |  |
| AQ ✻ Volatility |  | 19.166 |  | 1 |  | 4659.774 |  | < .001 |  |
| AQ ✻ Variability ✻ Volatility |  | 4.273 |  | 1 |  | 4661.029 |  | 0.039 |  |
| Note. Satterthwaite method for degrees of freedom | | | | | | | | | |
|  | | | | | | | | | |

| Fixed Effects Parameter Estimates | | | | | | | | | | | | | | | | | | | | | | | | | | | |
| --- | --- | --- | --- | --- | --- | --- | --- | --- | --- | --- | --- | --- | --- | --- | --- | --- | --- | --- | --- | --- | --- | --- | --- | --- | --- | --- | --- |
|  | | | | | | | | | | | | | | | | **95% Confidence Interval** | | | | | |  | | | | | |
| **Names** | | | | **Effect** | | | | **Estimate** | | | | **SE** | | | | **Lower** | | | | **Upper** | | **df** | | **t** | | **p** | |
| (Intercept) | | |  | (Intercept) | | |  | 14.082 | | |  | 0.355 | | |  | 13.386 | | |  | 14.778 |  | 36.998 |  | 39.655 |  | < .001 |  |
| nTurns | | |  | nTurns | | |  | 0.497 | | |  | 0.008 | | |  | 0.482 | | |  | 0.513 |  | 3842.454 |  | 61.965 |  | < .001 |  |
| AQ | | |  | AQ | | |  | 0.012 | | |  | 0.061 | | |  | -0.108 | | |  | 0.132 |  | 36.935 |  | 0.196 |  | 0.845 |  |
| Variability1 | | |  | 1 - 0 | | |  | 0.140 | | |  | 0.167 | | |  | -0.187 | | |  | 0.467 |  | 4684.260 |  | 0.839 |  | 0.401 |  |
| Volatility1 | | |  | 1 - 0 | | |  | -0.217 | | |  | 0.161 | | |  | -0.533 | | |  | 0.099 |  | 4660.627 |  | -1.344 |  | 0.179 |  |
| Variability1 ✻ Volatility1 | | |  | 1 - 0 ✻ 1 - 0 | | |  | -0.244 | | |  | 0.323 | | |  | -0.876 | | |  | 0.388 |  | 4660.975 |  | -0.758 |  | 0.449 |  |
| AQ ✻ Variability1 | | |  | AQ ✻ 1 - 0 | | |  | 0.037 | | |  | 0.028 | | |  | -0.016 | | |  | 0.091 |  | 4661.195 |  | 1.361 |  | 0.174 |  |
| AQ ✻ Volatility1 | | |  | AQ ✻ 1 - 0 | | |  | -0.120 | | |  | 0.027 | | |  | -0.174 | | |  | -0.066 |  | 4659.774 |  | -4.378 |  | < .001 |  |
| AQ ✻ Variability1 ✻ Volatility1 | | |  | AQ ✻ 1 - 0 ✻ 1 - 0 | | |  | 0.114 | | |  | 0.055 | | |  | 0.006 | | |  | 0.222 |  | 4661.029 |  | 2.067 |  | 0.039 |  |
|  | | | | | | | | | | | | | | | | | | | | | | | | | | | |
| Random Components | | | | | | | | | | | | | | | | | |  |  |  |  |  |  |  |  |  |  |
|  |  |  | | |  |  | | |  |  | | |  |  | | |  |  |  |  |  |  |  |  |  |  |  |
| **Groups** | | **Name** | | | | **SD** | | | | **Variance** | | | | **ICC** | | | |  |  |  |  |  |  |  |  |  |  |
| id |  | (Intercept) | | |  | 2.186 | | |  | 4.780 | | |  | 0.136 | | |  |  |  |  |  |  |  |  |  |  |  |
| Residual |  |  | | |  | 5.520 | | |  | 30.470 | | |  |  | | |  |  |  |  |  |  |  |  |  |  |  |
| Note. Number of Obs: 4707 , groups: id , 40 | | | | | | | | | | | | | | | | | |  |  |  |  |  |  |  |  |  |  |
|  | | | | | | | | | | | | | | | | | |  |  |  |  |  |  |  |  |  |  |

 Simple Effects

| Simple effects of Volatility : Omnibus Tests | | | | | | | | | | | |  |  |  |  |  |  |  |  |  |  |
| --- | --- | --- | --- | --- | --- | --- | --- | --- | --- | --- | --- | --- | --- | --- | --- | --- | --- | --- | --- | --- | --- |
| **Moderator levels** | |  | | | | | | | | | |  |  |  |  |  |  |  |  |  |  |
| **AQ** | | **X²** | | | **df** | | | **p** | | | |  |  |  |  |  |  |  |  |  |  |
| Mean-1·SD |  | 4.597 | |  | 1.000 | |  | 0.032 | | |  |  |  |  |  |  |  |  |  |  |  |
| Mean |  | 1.807 | |  | 1.000 | |  | 0.179 | | |  |  |  |  |  |  |  |  |  |  |  |
| Mean+1·SD |  | 16.367 | |  | 1.000 | |  | < .001 | | |  |  |  |  |  |  |  |  |  |  |  |
|  | | | | | | | | | | | |  |  |  |  |  |  |  |  |  |  |
| Simple effects of Volatility : Parameter estimates | | | | | | | | | | | | | | | | | | | | | |
| **Moderator levels** | |  | | | | | | | | | | | | **95% Confidence Interval** | | | |  | | | |
| **AQ** | | **contrast** | | | | **Estimate** | | | | **SE** | | | | **Lower** | | **Upper** | | **z** | | **p** | |
| Mean-1·SD |  | 1 - 0 |  | | | 0.489 | | |  | 0.228 | | |  | 0.042 |  | 0.935 |  | 2.144 |  | 0.032 |  |
| Mean |  | 1 - 0 |  | | | -0.217 | | |  | 0.161 | | |  | -0.533 |  | 0.099 |  | -1.344 |  | 0.179 |  |
| Mean+1·SD |  | 1 - 0 |  | | | -0.922 | | |  | 0.228 | | |  | -1.369 |  | -0.475 |  | -4.046 |  | < .001 |  |
| Note. Simple effects are estimated keeping constant other independent variable(s) in the model | | | | | | | | | | | | | | | | | | | | | |
|  | | | | | | | | | | | | | | | | | | | | | |

## Number of Hypothesis Switches Mixed Model

Mixed Model

| Model Info | | | |
| --- | --- | --- | --- |
|  |  |  |  |
| **Info** | |  | |
| Estimate |  | Linear mixed model fit by REML |  |
| Call |  | nHypSwitches ~ 1 + Variability + Volatility + AQ + Variability:Volatility + AQ:Volatility + AQ:Variability + AQ:Volatility:Variability+( 1 \| id ) |  |
| AIC |  | 36948.652 |  |
| R-squared Marginal |  | 0.020 |  |
| R-squared Conditional |  | 0.578 |  |
|  | | | |

Model Results

| Fixed Effect Omnibus tests | | | | | | | | | |
| --- | --- | --- | --- | --- | --- | --- | --- | --- | --- |
|  |  |  |  |  |  |  |  |  |  |
|  | | **F** | | **Num df** | | **Den df** | | **p** | |
| Variability |  | 195.913 |  | 1 |  | 4661.203 |  | < .001 |  |
| Volatility |  | 2.045 |  | 1 |  | 4661.206 |  | 0.153 |  |
| AQ |  | 0.116 |  | 1 |  | 38.005 |  | 0.736 |  |
| Variability ✻ Volatility |  | 6.446 |  | 1 |  | 4661.209 |  | 0.011 |  |
| Volatility ✻ AQ |  | 0.143 |  | 1 |  | 4661.099 |  | 0.705 |  |
| Variability ✻ AQ |  | 0.280 |  | 1 |  | 4661.234 |  | 0.597 |  |
| Variability ✻ Volatility ✻ AQ |  | 0.508 |  | 1 |  | 4661.207 |  | 0.476 |  |
| Note. Satterthwaite method for degrees of freedom | | | | | | | | | |
|  | | | | | | | | | |

| Fixed Effects Parameter Estimates | | | | | | | | | | | | | | | | | |
| --- | --- | --- | --- | --- | --- | --- | --- | --- | --- | --- | --- | --- | --- | --- | --- | --- | --- |
|  | | | | | | | | **95% Confidence Interval** | | | |  | | | | | |
| **Names** | | **Effect** | | **Estimate** | | **SE** | | **Lower** | | **Upper** | | **df** | | **t** | | **p** | |
| (Intercept) |  | (Intercept) |  | 42.218 |  | 2.187 |  | 37.932 |  | 46.505 |  | 38.013 |  | 19.303 |  | < .001 |  |
| Variability1 |  | 1 - 0 |  | -4.904 |  | 0.350 |  | -5.590 |  | -4.217 |  | 4661.203 |  | -13.997 |  | < .001 |  |
| Volatility1 |  | 1 - 0 |  | 0.501 |  | 0.350 |  | -0.186 |  | 1.188 |  | 4661.206 |  | 1.430 |  | 0.153 |  |
| AQ |  | AQ |  | -0.128 |  | 0.376 |  | -0.865 |  | 0.609 |  | 38.005 |  | -0.340 |  | 0.736 |  |
| Variability1 ✻ Volatility1 |  | 1 - 0 ✻ 1 - 0 |  | -1.779 |  | 0.701 |  | -3.152 |  | -0.406 |  | 4661.209 |  | -2.539 |  | 0.011 |  |
| Volatility1 ✻ AQ |  | 1 - 0 ✻ AQ |  | -0.023 |  | 0.060 |  | -0.140 |  | 0.094 |  | 4661.099 |  | -0.379 |  | 0.705 |  |
| Variability1 ✻ AQ |  | 1 - 0 ✻ AQ |  | -0.032 |  | 0.060 |  | -0.149 |  | 0.086 |  | 4661.234 |  | -0.529 |  | 0.597 |  |
| Variability1 ✻ Volatility1 ✻ AQ |  | 1 - 0 ✻ 1 - 0 ✻ AQ |  | 0.085 |  | 0.120 |  | -0.149 |  | 0.320 |  | 4661.207 |  | 0.712 |  | 0.476 |  |
|  | | | | | | | | | | | | | | | | | |

| Random Components | | | | | | | | | |
| --- | --- | --- | --- | --- | --- | --- | --- | --- | --- |
|  |  |  |  |  |  |  |  |  |  |
| **Groups** | | **Name** | | **SD** | | **Variance** | | **ICC** | |
| id |  | (Intercept) |  | 13.785 |  | 190.038 |  | 0.569 |  |
| Residual |  |  |  | 11.997 |  | 143.936 |  |  |  |
| Note. Number of Obs: 4707 , groups: id , 40 | | | | | | | | | |
|  | | | | | | | | | |

Post Hoc Tests

| Post Hoc Comparisons - Variability | | | | | | | | | | | | | | | | | | | | | | | |  |  |  |  |  |  |
| --- | --- | --- | --- | --- | --- | --- | --- | --- | --- | --- | --- | --- | --- | --- | --- | --- | --- | --- | --- | --- | --- | --- | --- | --- | --- | --- | --- | --- | --- |
| **Comparison** | | | | | | | | | |  | | | | | | | | | | | | | |  |  |  |  |  |  |
| **Variability** | |  | | **Variability** | | | | | | **Difference** | | | | **SE** | | | **z** | | | **p_bonferroni_** | | | |  |  |  |  |  |  |
| 0 |  | - |  | 1 | |  | | | | 4.904 | |  | | 0.350 | |  | 13.997 | |  | < .001 | | |  |  |  |  |  |  |  |
|  | | | | | | | | | | | | | | | | | | | | | | | |  |  |  |  |  |  |
| Post Hoc Comparisons - Variability ✻ Volatility | | | | | | | | | | | | | | | | | | | | | | | | | | | | | |
| **Comparison** | | | | | | | | | | | | | | | | | |  | | | | | | | | | | | |
| **Variability** | | **Volatility** | | | | |  | | **Variability** | | | | **Volatility** | | | | | **Difference** | | | | **SE** | | | **z** | | | **p_bonferroni_** | |
| 0 |  | 0 | | |  | | - |  | 0 | |  | | 1 | |  | | | -1.390 | | |  | 0.498 | |  | -2.792 | |  | 0.031 |  |
| 0 |  | 0 | | |  | | - |  | 1 | |  | | 0 | |  | | | 4.014 | | |  | 0.499 | |  | 8.044 | |  | < .001 |  |
| 0 |  | 0 | | |  | | - |  | 1 | |  | | 1 | |  | | | 4.403 | | |  | 0.495 | |  | 8.896 | |  | < .001 |  |
| 0 |  | 1 | | |  | | - |  | 1 | |  | | 1 | |  | | | 5.793 | | |  | 0.492 | |  | 11.779 | |  | < .001 |  |
| 1 |  | 0 | | |  | | - |  | 0 | |  | | 1 | |  | | | -5.405 | | |  | 0.496 | |  | -10.897 | |  | < .001 |  |
| 1 |  | 0 | | |  | | - |  | 1 | |  | | 1 | |  | | | 0.388 | | |  | 0.493 | |  | 0.788 | |  | 1.000 |  |
|  | | | | | | | | | | | | | | | | | | | | | | | | | | | | | |
|  | | | | | | | | | | | | | | | | | | | | | | | | | |  |  |  |  |

## Average Prediction Error Mixed Model:

Mixed Model

| Model Info | | | |
| --- | --- | --- | --- |
|  |  |  |  |
| **Info** | |  | |
| Estimate |  | Linear mixed model fit by REML |  |
| Call |  | AvPE ~ 1 + Variability + Volatility + AQ + Accuracy + Variability:Volatility + AQ:Volatility + AQ:Variability + Variability:Accuracy + Volatility:Accuracy + AQ:Accuracy + AQ:Volatility:Variability + Variability:Volatility:Accuracy + AQ:Accuracy:Variability + AQ:Accuracy:Volatility:Variability+( 1 \| id ) |  |
| AIC |  | 26741.610 |  |
| R-squared Marginal |  | 0.102 |  |
| R-squared Conditional |  | 0.634 |  |
|  | | | |

Model Results

| Fixed Effect Omnibus tests | | | | | | | | | |
| --- | --- | --- | --- | --- | --- | --- | --- | --- | --- |
|  |  |  |  |  |  |  |  |  |  |
|  | | **F** | | **Num df** | | **Den df** | | **p** | |
| Variability |  | 284.0514 |  | 1 |  | 4653.2 |  | < .001 |  |
| Volatility |  | 0.5022 |  | 1 |  | 4653.2 |  | 0.479 |  |
| AQ |  | 1.7937 |  | 1 |  | 38.4 |  | 0.188 |  |
| Accuracy |  | 172.5723 |  | 1 |  | 4655.9 |  | < .001 |  |
| Variability ✻ Volatility |  | 0.2649 |  | 1 |  | 4653.3 |  | 0.607 |  |
| Volatility ✻ AQ |  | 1.2145 |  | 1 |  | 4653.2 |  | 0.271 |  |
| Variability ✻ AQ |  | 10.5796 |  | 1 |  | 4653.2 |  | 0.001 |  |
| Variability ✻ Accuracy |  | 14.1596 |  | 1 |  | 4653.4 |  | < .001 |  |
| Volatility ✻ Accuracy |  | 0.0374 |  | 1 |  | 4653.3 |  | 0.847 |  |
| AQ ✻ Accuracy |  | 0.5401 |  | 1 |  | 4655.4 |  | 0.462 |  |
| Variability ✻ Volatility ✻ AQ |  | 3.50e-5 |  | 1 |  | 4653.4 |  | 0.995 |  |
| Variability ✻ Volatility ✻ Accuracy |  | 6.6103 |  | 1 |  | 4653.5 |  | 0.010 |  |
| Variability ✻ AQ ✻ Accuracy |  | 0.3085 |  | 1 |  | 4653.4 |  | 0.579 |  |
| Variability ✻ Volatility ✻ AQ ✻ Accuracy |  | 1.0043 |  | 2 |  | 4653.3 |  | 0.366 |  |
| Note. Satterthwaite method for degrees of freedom | | | | | | | | | |
|  | | | | | | | | | |

| Fixed Effects Parameter Estimates | | | | | | | | | | | | | | | | | |
| --- | --- | --- | --- | --- | --- | --- | --- | --- | --- | --- | --- | --- | --- | --- | --- | --- | --- |
|  | | | | | | | | **95% Confidence Interval** | | | |  | | | | | |
| **Names** | | **Effect** | | **Estimate** | | **SE** | | **Lower** | | **Upper** | | **df** | | **t** | | **p** | |
| (Intercept) |  | (Intercept) |  | 11.1392 |  | 0.7766 |  | 9.6170 |  | 12.6614 |  | 38.4 |  | 14.343 |  | < .001 |  |
| Variability1 |  | 1 - 0 |  | 2.6838 |  | 0.1592 |  | 2.3717 |  | 2.9959 |  | 4653.2 |  | 16.854 |  | < .001 |  |
| Volatility1 |  | 1 - 0 |  | 0.1128 |  | 0.1592 |  | -0.1991 |  | 0.4247 |  | 4653.2 |  | 0.709 |  | 0.479 |  |
| AQ |  | AQ |  | -0.1788 |  | 0.1335 |  | -0.4405 |  | 0.0829 |  | 38.4 |  | -1.339 |  | 0.188 |  |
| Accuracy1 |  | 1 - 0 |  | -2.1419 |  | 0.1630 |  | -2.4614 |  | -1.8223 |  | 4655.9 |  | -13.137 |  | < .001 |  |
| Variability1 ✻ Volatility1 |  | 1 - 0 ✻ 1 - 0 |  | -0.1641 |  | 0.3188 |  | -0.7889 |  | 0.4607 |  | 4653.3 |  | -0.515 |  | 0.607 |  |
| Volatility1 ✻ AQ |  | 1 - 0 ✻ AQ |  | -0.0310 |  | 0.0281 |  | -0.0861 |  | 0.0241 |  | 4653.2 |  | -1.102 |  | 0.271 |  |
| Variability1 ✻ AQ |  | 1 - 0 ✻ AQ |  | -0.1043 |  | 0.0281 |  | -0.1594 |  | -0.0492 |  | 4653.2 |  | -3.708 |  | < .001 |  |
| Variability1 ✻ Accuracy1 |  | 1 - 0 ✻ 1 - 0 |  | 1.2002 |  | 0.3190 |  | 0.5751 |  | 1.8253 |  | 4653.4 |  | 3.763 |  | < .001 |  |
| Volatility1 ✻ Accuracy1 |  | 1 - 0 ✻ 1 - 0 |  | -0.0616 |  | 0.3186 |  | -0.6861 |  | 0.5629 |  | 4653.3 |  | -0.193 |  | 0.847 |  |
| AQ ✻ Accuracy1 |  | AQ ✻ 1 - 0 |  | 0.0211 |  | 0.0287 |  | -0.0351 |  | 0.0773 |  | 4655.4 |  | 0.735 |  | 0.462 |  |
| Variability1 ✻ Volatility1 ✻ AQ |  | 1 - 0 ✻ 1 - 0 ✻ AQ |  | 0.0270 |  | 0.0563 |  | -0.0834 |  | 0.1374 |  | 4653.4 |  | 0.479 |  | 0.632 |  |
| Variability1 ✻ Volatility1 ✻ Accuracy1 |  | 1 - 0 ✻ 1 - 0 ✻ 1 - 0 |  | -1.6411 |  | 0.6383 |  | -2.8921 |  | -0.3900 |  | 4653.5 |  | -2.571 |  | 0.010 |  |
| Variability1 ✻ AQ ✻ Accuracy1 |  | 1 - 0 ✻ AQ ✻ 1 - 0 |  | -0.0183 |  | 0.0563 |  | -0.1286 |  | 0.0919 |  | 4653.2 |  | -0.326 |  | 0.744 |  |
| Variability0 ✻ Volatility1 ✻ AQ ✻ Accuracy1 |  | Variability0 ✻ 1 - 0 ✻ AQ ✻ 1 - 0 |  | 0.0405 |  | 0.0887 |  | -0.1333 |  | 0.2143 |  | 4653.4 |  | 0.457 |  | 0.648 |  |
| Variability1 ✻ Volatility1 ✻ AQ ✻ Accuracy1 |  | Variability1 ✻ 1 - 0 ✻ AQ ✻ 1 - 0 |  | 0.0932 |  | 0.0695 |  | -0.0429 |  | 0.2294 |  | 4653.2 |  | 1.342 |  | 0.180 |  |
|  | | | | | | | | | | | | | | | | | |

| Random Components | | | | | | | | | |
| --- | --- | --- | --- | --- | --- | --- | --- | --- | --- |
|  |  |  |  |  |  |  |  |  |  |
| **Groups** | | **Name** | | **SD** | | **Variance** | | **ICC** | |
| id |  | (Intercept) |  | 4.88 |  | 23.9 |  | 0.593 |  |
| Residual |  |  |  | 4.05 |  | 16.4 |  |  |  |
| Note. Number of Obs: 4707 , groups: id , 40 | | | | | | | | | |
|  | | | | | | | | | |

Post Hoc Tests

| Post Hoc Comparisons - Variability | | | | | | | | | | | | | |
| --- | --- | --- | --- | --- | --- | --- | --- | --- | --- | --- | --- | --- | --- |
| **Comparison** | | | | | |  | | | | | | | |
| **Variability** | |  | | **Variability** | | **Difference** | | **SE** | | **z** | | **p_bonferroni_** | |
| 0 |  | - |  | 1 |  | -2.68 |  | 0.159 |  | -16.9 |  | < .001 |  |
|  | | | | | | | | | | | | | |

Simple Effects

| Simple effects of Variability : Omnibus Tests | | | | | | | |
| --- | --- | --- | --- | --- | --- | --- | --- |
| **Moderator levels** | |  | | | | | |
| **AQ** | | **X²** | | **df** | | **p** | |
| Mean-1·SD |  | 208.5 |  | 1.00 |  | < .001 |  |
| Mean |  | 284.1 |  | 1.00 |  | < .001 |  |
| Mean+1·SD |  | 81.2 |  | 1.00 |  | < .001 |  |
|  | | | | | | | |

| Simple effects of Variability : Parameter estimates | | | | | | | | | | | | | | | |
| --- | --- | --- | --- | --- | --- | --- | --- | --- | --- | --- | --- | --- | --- | --- | --- |
| **Moderator levels** | |  | | | | | | **95% Confidence Interval** | | | |  | | | |
| **AQ** | | **contrast** | | **Estimate** | | **SE** | | **Lower** | | **Upper** | | **z** | | **p** | |
| Mean-1·SD |  | 1 - 0 |  | 3.29 |  | 0.228 |  | 2.85 |  | 3.74 |  | 14.44 |  | < .001 |  |
| Mean |  | 1 - 0 |  | 2.68 |  | 0.159 |  | 2.37 |  | 3.00 |  | 16.85 |  | < .001 |  |
| Mean+1·SD |  | 1 - 0 |  | 2.07 |  | 0.230 |  | 1.62 |  | 2.52 |  | 9.01 |  | < .001 |  |
| Note. Simple effects are estimated setting higher order moderator (if any) in covariates to zero and averaging across moderating factors levels (if any) | | | | | | | | | | | | | | | |
|  | | | | | | | | | | | | | | | |

## Condition-wise Prediction Error Slope Mixed Model: Accuracy Not Included

Mixed Model

| Model Info | | | |
| --- | --- | --- | --- |
|  |  |  |  |
| **Info** | |  | |
| Estimate |  | Linear mixed model fit by REML |  |
| Call |  | ConditionAvPEGradient ~ 1 + Variability + Volatility + AQ + Variability:Volatility + Variability:AQ + Volatility:AQ + Variability:Volatility:AQ+( 1 \| id ) |  |
| AIC |  | -1131.354 |  |
| R-squared Marginal |  | 0.104 |  |
| R-squared Conditional |  | 0.749 |  |
|  | | | |

Model Results

| Fixed Effect Omnibus tests | | | | | | | | | |
| --- | --- | --- | --- | --- | --- | --- | --- | --- | --- |
|  |  |  |  |  |  |  |  |  |  |
|  | | **F** | | **Num df** | | **Den df** | | **p** | |
| Variability |  | 58.154 |  | 1 |  | 114.000 |  | < .001 |  |
| Volatility |  | 3.959 |  | 1 |  | 114.000 |  | 0.049 |  |
| AQ |  | 0.260 |  | 1 |  | 38.000 |  | 0.613 |  |
| Variability ✻ Volatility |  | 0.217 |  | 1 |  | 114.000 |  | 0.643 |  |
| Variability ✻ AQ |  | 0.779 |  | 1 |  | 114.000 |  | 0.379 |  |
| Volatility ✻ AQ |  | 0.052 |  | 1 |  | 114.000 |  | 0.819 |  |
| Variability ✻ Volatility ✻ AQ |  | 0.020 |  | 1 |  | 114.000 |  | 0.887 |  |
| Note. Satterthwaite method for degrees of freedom | | | | | | | | | |
|  | | | | | | | | | |

| Fixed Effects Parameter Estimates | | | | | | | | | | | | | | | | | |
| --- | --- | --- | --- | --- | --- | --- | --- | --- | --- | --- | --- | --- | --- | --- | --- | --- | --- |
|  | | | | | | | | **95% Confidence Interval** | | | |  | | | | | |
| **Names** | | **Effect** | | **Estimate** | | **SE** | | **Lower** | | **Upper** | | **df** | | **t** | | **p** | |
| (Intercept) |  | (Intercept) |  | 4.350e-4 |  | 9.956e-4 |  | -0.002 |  | 0.002 |  | 38.000 |  | 0.437 |  | 0.665 |  |
| Variability1 |  | 1 - 0 |  | 0.005 |  | 5.923e-4 |  | 0.003 |  | 0.006 |  | 114.000 |  | 7.626 |  | < .001 |  |
| Volatility1 |  | 1 - 0 |  | -0.001 |  | 5.923e-4 |  | -0.002 |  | -1.762e−5 |  | 114.000 |  | -1.990 |  | 0.049 |  |
| AQ |  | AQ |  | 8.719e-5 |  | 1.711e-4 |  | -2.482e−4 |  | 4.226e-4 |  | 38.000 |  | 0.509 |  | 0.613 |  |
| Variability1 ✻ Volatility1 |  | 1 - 0 ✻ 1 - 0 |  | 5.514e-4 |  | 0.001 |  | -0.002 |  | 0.003 |  | 114.000 |  | 0.465 |  | 0.643 |  |
| Variability1 ✻ AQ |  | 1 - 0 ✻ AQ |  | -8.987e−5 |  | 1.018e-4 |  | -2.894e−4 |  | 1.097e-4 |  | 114.000 |  | -0.883 |  | 0.379 |  |
| Volatility1 ✻ AQ |  | 1 - 0 ✻ AQ |  | -2.329e−5 |  | 1.018e-4 |  | -2.228e−4 |  | 1.763e-4 |  | 114.000 |  | -0.229 |  | 0.819 |  |
| Variability1 ✻ Volatility1 ✻ AQ |  | 1 - 0 ✻ 1 - 0 ✻ AQ |  | -2.900e−5 |  | 2.036e-4 |  | -4.281e−4 |  | 3.701e-4 |  | 114.000 |  | -0.142 |  | 0.887 |  |
|  | | | | | | | | | | | | | | | | | |

| Random Components | | | | | | | | | |
| --- | --- | --- | --- | --- | --- | --- | --- | --- | --- |
|  |  |  |  |  |  |  |  |  |  |
| **Groups** | | **Name** | | **SD** | | **Variance** | | **ICC** | |
| id |  | (Intercept) |  | 0.006 |  | 3.614e-5 |  | 0.720 |  |
| Residual |  |  |  | 0.004 |  | 1.403e-5 |  |  |  |
| Note. Number of Obs: 160 , groups: id , 40 | | | | | | | | | |
|  | | | | | | | | | |

Post Hoc Tests

| Post Hoc Comparisons - Variability | | | | | | | | | | | | | | | |
| --- | --- | --- | --- | --- | --- | --- | --- | --- | --- | --- | --- | --- | --- | --- | --- |
| **Comparison** | | | | | |  | | | | | | | | | |
| **Variability** | |  | | **Variability** | | **Difference** | | **SE** | | **t** | | **df** | | **p_bonferroni_** | |
| 0 |  | - |  | 1 |  | -0.005 |  | 5.923e-4 |  | -7.626 |  | 114.000 |  | < .001 |  |
|  | | | | | | | | | | | | | | | |

| Post Hoc Comparisons - Volatility | | | | | | | | | | | | | | | |
| --- | --- | --- | --- | --- | --- | --- | --- | --- | --- | --- | --- | --- | --- | --- | --- |
| **Comparison** | | | | | |  | | | | | | | | | |
| **Volatility** | |  | | **Volatility** | | **Difference** | | **SE** | | **t** | | **df** | | **p_bonferroni_** | |
| 0 |  | - |  | 1 |  | 0.001 |  | 5.923e-4 |  | 1.990 |  | 114.000 |  | 0.049 |  |
|  | | | | | | | | | | | | | | | |

## Condition-wise Prediction Error Mixed Model: Accuracy Random Effect Included

|  |
| --- |

 Mixed Model

| Model Info | | | |
| --- | --- | --- | --- |
|  |  |  |  |
| **Info** | |  | |
| Estimate |  | Linear mixed model fit by REML |  |
| Call |  | ConditionAvPEGradient ~ 1 + Variability + Volatility + AQ + Variability:Volatility + Variability:AQ + Volatility:AQ + Variability:Volatility:AQ+( 1 \| id )+( 1 \| Accuracy ) |  |
| AIC |  | -1214.8500 |  |
| R-squared Marginal |  | 0.0936 |  |
| R-squared Conditional |  | 0.7798 |  |
|  | | | |

Model Results

| Fixed Effect Omnibus tests | | | | | | | | | |
| --- | --- | --- | --- | --- | --- | --- | --- | --- | --- |
|  |  |  |  |  |  |  |  |  |  |
|  | | **F** | | **Num df** | | **Den df** | | **p** | |
| Variability |  | 1.2907 |  | 1 |  | 2.19e-9 |  | 1.000 |  |
| Volatility |  | 4.2748 |  | 1 |  | 113.1 |  | 0.041 |  |
| AQ |  | 0.2722 |  | 1 |  | 38.0 |  | 0.605 |  |
| Variability ✻ Volatility |  | 0.3029 |  | 1 |  | 113.1 |  | 0.583 |  |
| Variability ✻ AQ |  | 0.8424 |  | 1 |  | 113.0 |  | 0.361 |  |
| Volatility ✻ AQ |  | 0.0367 |  | 1 |  | 113.0 |  | 0.848 |  |
| Variability ✻ Volatility ✻ AQ |  | 0.0320 |  | 1 |  | 113.0 |  | 0.858 |  |
|  | | | | | | | | | |

| Fixed Effects Parameter Estimates | | | | | | | | | | | | | | | | | |
| --- | --- | --- | --- | --- | --- | --- | --- | --- | --- | --- | --- | --- | --- | --- | --- | --- | --- |
|  | | | | | | | | **95% Confidence Interval** | | | |  | | | | | |
| **Names** | | **Effect** | | **Estimate** | | **SE** | | **Lower** | | **Upper** | | **df** | | **t** | | **p** | |
| (Intercept) |  | (Intercept) |  | 4.10e-4 |  | 0.00222 |  | -0.00395 |  | 0.00477 |  | 3.27e-9 |  | 0.185 |  | 1.000 |  |
| Variability1 |  | 1 - 0 |  | 0.00457 |  | 0.00402 |  | -0.00331 |  | 0.01245 |  | 2.19e-9 |  | 1.136 |  | 1.000 |  |
| Volatility1 |  | 1 - 0 |  | -0.00123 |  | 5.95e-4 |  | -0.00240 |  | -6.41e−5 |  | 113.1 |  | -2.068 |  | 0.041 |  |
| AQ |  | AQ |  | 8.91e-5 |  | 1.71e-4 |  | -2.46e−4 |  | 4.24e-4 |  | 38.0 |  | 0.522 |  | 0.605 |  |
| Variability1 ✻ Volatility1 |  | 1 - 0 ✻ 1 - 0 |  | 6.55e-4 |  | 0.00119 |  | -0.00168 |  | 0.00299 |  | 113.1 |  | 0.550 |  | 0.583 |  |
| Variability1 ✻ AQ |  | 1 - 0 ✻ AQ |  | -9.36e−5 |  | 1.02e-4 |  | -2.94e−4 |  | 1.06e-4 |  | 113.0 |  | -0.918 |  | 0.361 |  |
| Volatility1 ✻ AQ |  | 1 - 0 ✻ AQ |  | -1.95e−5 |  | 1.02e-4 |  | -2.19e−4 |  | 1.80e-4 |  | 113.0 |  | -0.192 |  | 0.848 |  |
| Variability1 ✻ Volatility1 ✻ AQ |  | 1 - 0 ✻ 1 - 0 ✻ AQ |  | -3.65e−5 |  | 2.04e-4 |  | -4.36e−4 |  | 3.63e-4 |  | 113.0 |  | -0.179 |  | 0.858 |  |
|  | | | | | | | | | | | | | | | | | |

| Random Components | | | | | | | | | |
| --- | --- | --- | --- | --- | --- | --- | --- | --- | --- |
|  |  |  |  |  |  |  |  |  |  |
| **Groups** | | **Name** | | **SD** | | **Variance** | | **ICC** | |
| id |  | (Intercept) |  | 0.00599 |  | 3.59e-5 |  | 0.719 |  |
| Accuracy |  | (Intercept) |  | 0.00281 |  | 7.91e-6 |  | 0.360 |  |
| Residual |  |  |  | 0.00375 |  | 1.41e-5 |  |  |  |
|  | | | | | | | | | |

| Random Effect LRT | | | | | | | | | | | |
| --- | --- | --- | --- | --- | --- | --- | --- | --- | --- | --- | --- |
|  |  |  |  |  |  |  |  |  |  |  |  |
| **Test** | | **N. par** | | **AIC** | | **LRT** | | **df** | | **p** | |
| (1 \| id) |  | 10 |  | -1003 |  | 100 |  | 1.00 |  | < .001 |  |
| (1 \| Accuracy) |  | 10 |  | -1103 |  | 0 |  | 1.00 |  | 1.000 |  |
|  | | | | | | | | | | | |

Post Hoc Tests

| Post Hoc Comparisons - Volatility | | | | | | | | | | | | | | | |
| --- | --- | --- | --- | --- | --- | --- | --- | --- | --- | --- | --- | --- | --- | --- | --- |
| **Comparison** | | | | | |  | | | | | | | | | |
| **Volatility** | |  | | **Volatility** | | **Difference** | | **SE** | | **t** | | **df** | | **p_bonferroni_** | |
| 0 |  | - |  | 1 |  | 0.00123 |  | 5.95e-4 |  | 2.07 |  | 113 |  | 0.041 |  |
|  | | | | | | | | | | | | | | | |

## Prediction Error Slope by Accuracy and Agency Mixed Model

Mixed Model

| Model Info | | | |
| --- | --- | --- | --- |
|  |  |  |  |
| **Info** | |  | |
| Estimate |  | Linear mixed model fit by REML |  |
| Call |  | AgAccAvPEGradient ~ 1 + JudgedAgency + Accuracy + AQ + JudgedAgency:Accuracy + Accuracy:AQ + JudgedAgency:AQ + Accuracy:JudgedAgency:AQ+( 1 \| id ) |  |
| AIC |  | -1083.147 |  |
| R-squared Marginal |  | 0.168 |  |
| R-squared Conditional |  | 0.746 |  |
|  | | | |

Model Results

| Fixed Effect Omnibus tests | | | | | | | | | |
| --- | --- | --- | --- | --- | --- | --- | --- | --- | --- |
|  |  |  |  |  |  |  |  |  |  |
|  | | **F** | | **Num df** | | **Den df** | | **p** | |
| JudgedAgency |  | 82.886 |  | 1 |  | 113.120 |  | < .001 |  |
| Accuracy |  | 1.279 |  | 1 |  | 113.120 |  | 0.260 |  |
| AQ |  | 0.001 |  | 1 |  | 38.007 |  | 0.971 |  |
| JudgedAgency ✻ Accuracy |  | 12.785 |  | 1 |  | 113.120 |  | < .001 |  |
| Accuracy ✻ AQ |  | 0.512 |  | 1 |  | 113.071 |  | 0.476 |  |
| JudgedAgency ✻ AQ |  | 0.398 |  | 1 |  | 113.071 |  | 0.529 |  |
| JudgedAgency ✻ Accuracy ✻ AQ |  | 5.684 |  | 1 |  | 113.071 |  | 0.019 |  |
| Note. Satterthwaite method for degrees of freedom | | | | | | | | | |
|  | | | | | | | | | |

| Fixed Effects Parameter Estimates | | | | | | | | | | | | | | | | | |
| --- | --- | --- | --- | --- | --- | --- | --- | --- | --- | --- | --- | --- | --- | --- | --- | --- | --- |
|  | | | | | | | | **95% Confidence Interval** | | | |  | | | | | |
| **Names** | | **Effect** | | **Estimate** | | **SE** | | **Lower** | | **Upper** | | **df** | | **t** | | **p** | |
| (Intercept) |  | (Intercept) |  | 0.004 |  | 0.001 |  | 0.001 |  | 0.006 |  | 38.057 |  | 3.285 |  | 0.002 |  |
| JudgedAgency1 |  | 1 - 0 |  | -0.006 |  | 6.892e-4 |  | -0.008 |  | -0.005 |  | 113.120 |  | -9.104 |  | < .001 |  |
| Accuracy1 |  | 1 - 0 |  | -7.794e−4 |  | 6.892e-4 |  | -0.002 |  | 5.714e-4 |  | 113.120 |  | -1.131 |  | 0.260 |  |
| AQ |  | AQ |  | -6.890e−6 |  | 1.873e-4 |  | -3.740e−4 |  | 3.602e-4 |  | 38.007 |  | -0.037 |  | 0.971 |  |
| JudgedAgency1 ✻ Accuracy1 |  | 1 - 0 ✻ 1 - 0 |  | -0.005 |  | 0.001 |  | -0.008 |  | -0.002 |  | 113.120 |  | -3.576 |  | < .001 |  |
| Accuracy1 ✻ AQ |  | 1 - 0 ✻ AQ |  | -8.447e−5 |  | 1.181e-4 |  | -3.159e−4 |  | 1.469e-4 |  | 113.071 |  | -0.715 |  | 0.476 |  |
| JudgedAgency1 ✻ AQ |  | 1 - 0 ✻ AQ |  | 7.449e-5 |  | 1.181e-4 |  | -1.569e−4 |  | 3.059e-4 |  | 113.071 |  | 0.631 |  | 0.529 |  |
| JudgedAgency1 ✻ Accuracy1 ✻ AQ |  | 1 - 0 ✻ 1 - 0 ✻ AQ |  | 5.630e-4 |  | 2.361e-4 |  | 1.002e-4 |  | 0.001 |  | 113.071 |  | 2.384 |  | 0.019 |  |
|  | | | | | | | | | | | | | | | | | |

| Random Components | | | | | | | | | |
| --- | --- | --- | --- | --- | --- | --- | --- | --- | --- |
|  |  |  |  |  |  |  |  |  |  |
| **Groups** | | **Name** | | **SD** | | **Variance** | | **ICC** | |
| id |  | (Intercept) |  | 0.007 |  | 4.277e-5 |  | 0.694 |  |
| Residual |  |  |  | 0.004 |  | 1.884e-5 |  |  |  |
| Note. Number of Obs: 159 , groups: id , 40 | | | | | | | | | |
|  | | | | | | | | | |

Post Hoc Tests

| Post Hoc Comparisons - JudgedAgency ✻ Accuracy | | | | | | | | | | | | | | | | | | | | | | | | | | | | | | | | | | | |
| --- | --- | --- | --- | --- | --- | --- | --- | --- | --- | --- | --- | --- | --- | --- | --- | --- | --- | --- | --- | --- | --- | --- | --- | --- | --- | --- | --- | --- | --- | --- | --- | --- | --- | --- | --- |
| **Comparison** | | | | | | | | | | | | | | | | | |  | | | | | | | | | | | | | | | | | |
| **JudgedAgency** | | | **Accuracy** | | | | |  | | **JudgedAgency** | | | | **Accuracy** | | | | **Difference** | | | | **SE** | | | | **t** | | | **df** | | | | **p_bonferroni_** | | |
| 0 |  | | 0 | | |  | | - |  | 0 | |  | | 1 | |  | | -0.002 | |  | | 9.707e-4 | | |  | -1.736 |  | | 113.001 | |  | | 0.512 | |  |
| 0 |  | | 0 | | |  | | - |  | 1 | |  | | 0 | |  | | 0.004 | |  | | 9.787e-4 | | |  | 3.893 |  | | 113.119 | |  | | 0.001 | |  |
| 0 |  | | 0 | | |  | | - |  | 1 | |  | | 1 | |  | | 0.007 | |  | | 9.707e-4 | | |  | 7.267 |  | | 113.001 | |  | | < .001 | |  |
| 0 |  | | 1 | | |  | | - |  | 1 | |  | | 1 | |  | | 0.009 | |  | | 9.707e-4 | | |  | 9.003 |  | | 113.001 | |  | | < .001 | |  |
| 1 |  | | 0 | | |  | | - |  | 0 | |  | | 1 | |  | | -0.005 | |  | | 9.787e-4 | | |  | -5.615 |  | | 113.119 | |  | | < .001 | |  |
| 1 |  | | 0 | | |  | | - |  | 1 | |  | | 1 | |  | | 0.003 | |  | | 9.787e-4 | | |  | 3.314 |  | | 113.119 | |  | | 0.007 | |  |
|  | | | | | | | | | | | | | | | | | | | | | | | | | | | | | | | | | | | |
| Post Hoc Comparisons - JudgedAgency | | | | | | | | | | | | | | | | | | | | | | | | | | | | | | | | | |  |  |
| **Comparison** | | | | | | | | | | | | |  | | | | | | | | | | | | | | | | | | | | |  |  |
| **JudgedAgency** | | | |  | | | **JudgedAgency** | | | | | | **Difference** | | | | **SE** | | | | **t** | | | **df** | | | | | | **p_bonferroni_** | | | |  |  |
| 0 | |  | | - |  | | 1 | | | |  | | 0.006 | |  | | 6.892e-4 | |  | | 9.104 | |  | 113.061 | | | |  | | < .001 | |  | |  |  |
|  | | | | | | | | | | | | | | | | | | | | | | | | | | | | | | | | | |  |  |

 Simple Effects

| Simple effects of Accuracy : Omnibus Tests | | | | | | | | | | | | | | | | | | | | | | | |  |  |  |  |  |  |  |  |
| --- | --- | --- | --- | --- | --- | --- | --- | --- | --- | --- | --- | --- | --- | --- | --- | --- | --- | --- | --- | --- | --- | --- | --- | --- | --- | --- | --- | --- | --- | --- | --- |
| **Moderator levels** | | | | | | | |  | | | | | | | | | | | | | | | |  |  |  |  |  |  |  |  |
| **JudgedAgency** | | | | **AQ** | | | | **F** | | | | **Num df** | | | | **Den df** | | | | **p** | | | |  |  |  |  |  |  |  |  |
| 0 | |  | | Mean-1·SD | | |  | 7.744 | | |  | 1.000 | | |  | 113.060 | | |  | 0.006 | | |  |  |  |  |  |  |  |  |  |
|  | |  | | Mean | | |  | 3.013 | | |  | 1.000 | | |  | 113.060 | | |  | 0.085 | | |  |  |  |  |  |  |  |  |  |
|  | |  | | Mean+1·SD | | |  | 0.110 | | |  | 1.000 | | |  | 113.060 | | |  | 0.741 | | |  |  |  |  |  |  |  |  |  |
| 1 | |  | | Mean-1·SD | | |  | 10.058 | | |  | 1.000 | | |  | 113.180 | | |  | 0.002 | | |  |  |  |  |  |  |  |  |  |
|  | |  | | Mean | | |  | 10.985 | | |  | 1.000 | | |  | 113.180 | | |  | 0.001 | | |  |  |  |  |  |  |  |  |  |
|  | |  | | Mean+1·SD | | |  | 2.294 | | |  | 1.000 | | |  | 113.080 | | |  | 0.133 | | |  |  |  |  |  |  |  |  |  |
|  | | | | | | | | | | | | | | | | | | | | | | | |  |  |  |  |  |  |  |  |
| Simple effects of Accuracy : Parameter estimates | | | | | | | | | | | | | | | | | | | | | | | | | | | | | | | |
| **Moderator levels** | | | | | |  | | | | | | | | | | | | **95% Confidence Interval** | | | | | | | |  | | | | | |
| **JudgedAgency** | | | **AQ** | | | **contrast** | | | | **Estimate** | | | | **SE** | | | | **Lower** | | | | **Upper** | | | | **df** | | **t** | | **p** | |
| 0 |  | | Mean-1·SD | |  | 1 - 0 | | |  | 0.004 | | |  | 0.001 | | |  | 0.001 | | |  | 0.007 | | |  | 113.060 |  | 2.783 |  | 0.006 |  |
|  |  | | Mean | |  | 1 - 0 | | |  | 0.002 | | |  | 9.707e-4 | | |  | -2.382e−4 | | |  | 0.004 | | |  | 113.060 |  | 1.736 |  | 0.085 |  |
|  |  | | Mean+1·SD | |  | 1 - 0 | | |  | -4.563e−4 | | |  | 0.001 | | |  | -0.003 | | |  | 0.002 | | |  | 113.060 |  | -0.331 |  | 0.741 |  |
| 1 |  | | Mean-1·SD | |  | 1 - 0 | | |  | -0.004 | | |  | 0.001 | | |  | -0.007 | | |  | -0.002 | | |  | 113.179 |  | -3.171 |  | 0.002 |  |
|  |  | | Mean | |  | 1 - 0 | | |  | -0.003 | | |  | 9.787e-4 | | |  | -0.005 | | |  | -0.001 | | |  | 113.178 |  | -3.314 |  | 0.001 |  |
|  |  | | Mean+1·SD | |  | 1 - 0 | | |  | -0.002 | | |  | 0.001 | | |  | -0.005 | | |  | 6.439e-4 | | |  | 113.080 |  | -1.515 |  | 0.133 |  |
|  | | | | | | | | | | | | | | | | | | | | | | | | | | | | | | | |

Estimated Marginal Means

| JudgedAgency | | | | | | | | | | | | | | | | | | | | | | | | | | | | | | |  |  |  |  |  |  |  |
| --- | --- | --- | --- | --- | --- | --- | --- | --- | --- | --- | --- | --- | --- | --- | --- | --- | --- | --- | --- | --- | --- | --- | --- | --- | --- | --- | --- | --- | --- | --- | --- | --- | --- | --- | --- | --- | --- |
|  | | | | | | | | | | | | | | | | | | | **95% Confidence Interval** | | | | | | | | | | | |  |  |  |  |  |  |  |
| **JudgedAgency** | | | | | | **Mean** | | | | **SE** | | | | **df** | | | | | **Lower** | | | | | **Upper** | | | | | | |  |  |  |  |  |  |  |
| 0 |  | | | | | 0.007 | |  | | 0.001 | | |  | 45.757 | | |  | | 0.004 | | | |  | 0.009 | | | | |  | |  |  |  |  |  |  |  |
| 1 |  | | | | | 4.434e-4 | |  | | 0.001 | | |  | 46.012 | | |  | | -0.002 | | | |  | 0.003 | | | | |  | |  |  |  |  |  |  |  |
| Note. Estimated means are estimated averaging across interacting variables | | | | | | | | | | | | | | | | | | | | | | | | | | | | | | |  |  |  |  |  |  |  |
|  | | | | | | | | | | | | | | | | | | | | | | | | | | | | | | |  |  |  |  |  |  |  |
| Accuracy | | | | | | | | | | | | | | | | | | | | | | | | | | | | | | | | | |  |  |  |  |
|  | | | | | | | | | | | | | | | | | | | | **95% Confidence Interval** | | | | | | | | | | | | | |  |  |  |  |
| **Accuracy** | | | | **Mean** | | | | | **SE** | | | | | | **df** | | | | | **Lower** | | | | | | | | **Upper** | | | | | |  |  |  |  |
| 0 | |  | | 0.004 | | |  | | 0.001 | | |  | | | 46.012 | | |  | | 0.002 | | | | | |  | | 0.006 | | | |  | |  |  |  |  |
| 1 | |  | | 0.003 | | |  | | 0.001 | | |  | | | 45.757 | | |  | | 8.915e-4 | | | | | |  | | 0.005 | | | |  | |  |  |  |  |
| Note. Estimated means are estimated averaging across interacting variables | | | | | | | | | | | | | | | | | | | | | | | | | | | | | | | | | |  |  |  |  |
|  | | | | | | | | | | | | | | | | | | | | | | | | | | | | | | | | | |  |  |  |  |
| Accuracy:JudgedAgency | | | | | | | | | | | | | | | | | | | | | | | | | | | | | | | | | | | | | |
|  | | | | | | | | | | | | | | | | | | | | | | | | | | | | | | | | | **95% Confidence Interval** | | | | |
| **Accuracy** | | | | | **JudgedAgency** | | | | | | | | | | | **Mean** | | | | | | **SE** | | | | | **df** | | | | | | **Lower** | | | **Upper** | |
| 0 | | |  | | 0 | | | | | |  | | | | | 0.006 | | | | |  | 0.001 | | |  | | 62.125 | | |  | | | 0.003 | |  | 0.008 |  |
| 1 | | |  | | 0 | | | | | |  | | | | | 0.008 | | | | |  | 0.001 | | |  | | 62.125 | | |  | | | 0.005 | |  | 0.010 |  |
| 0 | | |  | | 1 | | | | | |  | | | | | 0.002 | | | | |  | 0.001 | | |  | | 63.180 | | |  | | | -4.272e−4 | |  | 0.005 |  |
| 1 | | |  | | 1 | | | | | |  | | | | | -0.001 | | | | |  | 0.001 | | |  | | 62.125 | | |  | | | -0.004 | |  | 0.001 |  |
| Note. Estimated means are estimated keeping constant other independent variable(s) in the model to the mean | | | | | | | | | | | | | | | | | | | | | | | | | | | | | | | | | | | | | |
|  | | | | | | | | | | | | | | | | | | | | | | | | | | | | | | | | | | | | | |

## Volatility ERPE Mixed Model

Mixed Model

| Model Info | | | |
| --- | --- | --- | --- |
|  |  |  |  |
| **Info** | |  | |
| Estimate |  | Linear mixed model fit by REML |  |
| Call |  | VolSwitchERPE ~ 1 + avPE + Volatility + Variability + AQ + TimeBin + Volatility:Variability + Volatility:TimeBin + Variability:TimeBin + AQ:TimeBin + AQ:Variability + AQ:Volatility + Volatility:Variability:TimeBin + AQ:TimeBin:Variability + AQ:TimeBin:Volatility + AQ:Variability:Volatility + AQ:TimeBin:Variability:Volatility+( 1 \| id ) |  |
| AIC |  | 2504.522 |  |
| R-squared Marginal |  | 0.962 |  |
| R-squared Conditional |  | 0.964 |  |
|  | | | |

Model Results

| Fixed Effect Omnibus tests | | | | | | | | | |
| --- | --- | --- | --- | --- | --- | --- | --- | --- | --- |
|  |  |  |  |  |  |  |  |  |  |
|  | | **F** | | **Num df** | | **Den df** | | **p** | |
| avPE |  | 10716.298 |  | 1 |  | 51.346 |  | < .001 |  |
| Volatility |  | 0.520 |  | 1 |  | 722.053 |  | 0.471 |  |
| Variability |  | 3.909 |  | 1 |  | 662.430 |  | 0.048 |  |
| AQ |  | 0.286 |  | 1 |  | 37.521 |  | 0.596 |  |
| TimeBin |  | 0.155 |  | 4 |  | 721.814 |  | 0.961 |  |
| Volatility ✻ Variability |  | 0.406 |  | 1 |  | 722.935 |  | 0.524 |  |
| Volatility ✻ TimeBin |  | 1.000 |  | 4 |  | 721.814 |  | 0.407 |  |
| Variability ✻ TimeBin |  | 0.293 |  | 4 |  | 721.814 |  | 0.883 |  |
| AQ ✻ TimeBin |  | 0.067 |  | 4 |  | 721.814 |  | 0.992 |  |
| Variability ✻ AQ |  | 0.516 |  | 1 |  | 729.816 |  | 0.473 |  |
| Volatility ✻ AQ |  | 2.289 |  | 1 |  | 721.995 |  | 0.131 |  |
| Volatility ✻ Variability ✻ TimeBin |  | 0.572 |  | 4 |  | 721.814 |  | 0.683 |  |
| Variability ✻ AQ ✻ TimeBin |  | 0.533 |  | 4 |  | 721.814 |  | 0.712 |  |
| Volatility ✻ AQ ✻ TimeBin |  | 0.360 |  | 4 |  | 721.814 |  | 0.837 |  |
| Volatility ✻ Variability ✻ AQ |  | 0.157 |  | 1 |  | 722.043 |  | 0.692 |  |
| Volatility ✻ Variability ✻ AQ ✻ TimeBin |  | 0.095 |  | 4 |  | 721.814 |  | 0.984 |  |
|  | | | | | | | | | |

| Fixed Effects Parameter Estimates | | | | | | | | | | | | | | | | | |
| --- | --- | --- | --- | --- | --- | --- | --- | --- | --- | --- | --- | --- | --- | --- | --- | --- | --- |
|  | | | | | | | | **95% Confidence Interval** | | | |  | | | | | |
| **Names** | | **Effect** | | **Estimate** | | **SE** | | **Lower** | | **Upper** | | **df** | | **t** | | **p** | |
| (Intercept) |  | (Intercept) |  | 10.726 |  | 0.051 |  | 10.626 |  | 10.826 |  | 37.115 |  | 210.753 |  | < .001 |  |
| avPE |  | avPE |  | 1.010 |  | 0.010 |  | 0.991 |  | 1.029 |  | 51.346 |  | 103.520 |  | < .001 |  |
| Volatility1 |  | 2 - 1 |  | 0.054 |  | 0.076 |  | -0.094 |  | 0.203 |  | 722.053 |  | 0.721 |  | 0.471 |  |
| Variability1 |  | 2 - 1 |  | 0.162 |  | 0.082 |  | 0.001 |  | 0.324 |  | 662.430 |  | 1.977 |  | 0.048 |  |
| AQ |  | AQ |  | -0.005 |  | 0.009 |  | -0.022 |  | 0.013 |  | 37.521 |  | -0.535 |  | 0.596 |  |
| TimeBin1 |  | 2 - 1 |  | 0.025 |  | 0.119 |  | -0.209 |  | 0.259 |  | 721.814 |  | 0.209 |  | 0.834 |  |
| TimeBin2 |  | 3 - 1 |  | 0.017 |  | 0.119 |  | -0.217 |  | 0.251 |  | 721.814 |  | 0.142 |  | 0.887 |  |
| TimeBin3 |  | 4 - 1 |  | -0.046 |  | 0.119 |  | -0.280 |  | 0.188 |  | 721.814 |  | -0.387 |  | 0.699 |  |
| TimeBin4 |  | 5 - 1 |  | 0.041 |  | 0.119 |  | -0.194 |  | 0.275 |  | 721.814 |  | 0.340 |  | 0.734 |  |
| Volatility1 ✻ Variability1 |  | 2 - 1 ✻ 2 - 1 |  | -0.096 |  | 0.151 |  | -0.393 |  | 0.200 |  | 722.935 |  | -0.637 |  | 0.524 |  |
| Volatility1 ✻ TimeBin1 |  | 2 - 1 ✻ 2 - 1 |  | 0.028 |  | 0.239 |  | -0.440 |  | 0.496 |  | 721.814 |  | 0.116 |  | 0.907 |  |
| Volatility1 ✻ TimeBin2 |  | 2 - 1 ✻ 3 - 1 |  | -0.265 |  | 0.239 |  | -0.734 |  | 0.203 |  | 721.814 |  | -1.110 |  | 0.267 |  |
| Volatility1 ✻ TimeBin3 |  | 2 - 1 ✻ 4 - 1 |  | -0.348 |  | 0.239 |  | -0.816 |  | 0.120 |  | 721.814 |  | -1.456 |  | 0.146 |  |
| Volatility1 ✻ TimeBin4 |  | 2 - 1 ✻ 5 - 1 |  | -0.048 |  | 0.239 |  | -0.517 |  | 0.420 |  | 721.814 |  | -0.202 |  | 0.840 |  |
| Variability1 ✻ TimeBin1 |  | 2 - 1 ✻ 2 - 1 |  | -0.013 |  | 0.239 |  | -0.481 |  | 0.455 |  | 721.814 |  | -0.054 |  | 0.957 |  |
| Variability1 ✻ TimeBin2 |  | 2 - 1 ✻ 3 - 1 |  | 0.203 |  | 0.239 |  | -0.265 |  | 0.671 |  | 721.814 |  | 0.850 |  | 0.395 |  |
| Variability1 ✻ TimeBin3 |  | 2 - 1 ✻ 4 - 1 |  | 0.103 |  | 0.239 |  | -0.366 |  | 0.571 |  | 721.814 |  | 0.430 |  | 0.667 |  |
| Variability1 ✻ TimeBin4 |  | 2 - 1 ✻ 5 - 1 |  | 0.013 |  | 0.239 |  | -0.455 |  | 0.482 |  | 721.814 |  | 0.056 |  | 0.956 |  |
| AQ ✻ TimeBin1 |  | AQ ✻ 2 - 1 |  | -0.007 |  | 0.021 |  | -0.048 |  | 0.033 |  | 721.814 |  | -0.362 |  | 0.718 |  |
| AQ ✻ TimeBin2 |  | AQ ✻ 3 - 1 |  | -0.008 |  | 0.021 |  | -0.049 |  | 0.032 |  | 721.814 |  | -0.408 |  | 0.683 |  |
| AQ ✻ TimeBin3 |  | AQ ✻ 4 - 1 |  | -0.005 |  | 0.021 |  | -0.045 |  | 0.036 |  | 721.814 |  | -0.223 |  | 0.824 |  |
| AQ ✻ TimeBin4 |  | AQ ✻ 5 - 1 |  | -0.009 |  | 0.021 |  | -0.049 |  | 0.031 |  | 721.814 |  | -0.450 |  | 0.653 |  |
| Variability1 ✻ AQ |  | 2 - 1 ✻ AQ |  | -0.009 |  | 0.013 |  | -0.035 |  | 0.016 |  | 729.816 |  | -0.718 |  | 0.473 |  |
| Volatility1 ✻ AQ |  | 2 - 1 ✻ AQ |  | 0.020 |  | 0.013 |  | -0.006 |  | 0.045 |  | 721.995 |  | 1.513 |  | 0.131 |  |
| Volatility1 ✻ Variability1 ✻ TimeBin1 |  | 2 - 1 ✻ 2 - 1 ✻ 2 - 1 |  | 0.356 |  | 0.478 |  | -0.581 |  | 1.293 |  | 721.814 |  | 0.745 |  | 0.456 |  |
| Volatility1 ✻ Variability1 ✻ TimeBin2 |  | 2 - 1 ✻ 2 - 1 ✻ 3 - 1 |  | 0.134 |  | 0.478 |  | -0.802 |  | 1.071 |  | 721.814 |  | 0.281 |  | 0.779 |  |
| Volatility1 ✻ Variability1 ✻ TimeBin3 |  | 2 - 1 ✻ 2 - 1 ✻ 4 - 1 |  | 0.075 |  | 0.478 |  | -0.861 |  | 1.012 |  | 721.814 |  | 0.158 |  | 0.875 |  |
| Volatility1 ✻ Variability1 ✻ TimeBin4 |  | 2 - 1 ✻ 2 - 1 ✻ 5 - 1 |  | 0.630 |  | 0.478 |  | -0.307 |  | 1.566 |  | 721.814 |  | 1.318 |  | 0.188 |  |
| Variability1 ✻ AQ ✻ TimeBin1 |  | 2 - 1 ✻ AQ ✻ 2 - 1 |  | -0.044 |  | 0.041 |  | -0.124 |  | 0.036 |  | 721.814 |  | -1.071 |  | 0.284 |  |
| Variability1 ✻ AQ ✻ TimeBin2 |  | 2 - 1 ✻ AQ ✻ 3 - 1 |  | -0.005 |  | 0.041 |  | -0.085 |  | 0.076 |  | 721.814 |  | -0.114 |  | 0.909 |  |
| Variability1 ✻ AQ ✻ TimeBin3 |  | 2 - 1 ✻ AQ ✻ 4 - 1 |  | 0.012 |  | 0.041 |  | -0.069 |  | 0.092 |  | 721.814 |  | 0.291 |  | 0.771 |  |
| Variability1 ✻ AQ ✻ TimeBin4 |  | 2 - 1 ✻ AQ ✻ 5 - 1 |  | -0.016 |  | 0.041 |  | -0.096 |  | 0.065 |  | 721.814 |  | -0.381 |  | 0.703 |  |
| Volatility1 ✻ AQ ✻ TimeBin1 |  | 2 - 1 ✻ AQ ✻ 2 - 1 |  | 0.007 |  | 0.041 |  | -0.073 |  | 0.088 |  | 721.814 |  | 0.171 |  | 0.865 |  |
| Volatility1 ✻ AQ ✻ TimeBin2 |  | 2 - 1 ✻ AQ ✻ 3 - 1 |  | 0.031 |  | 0.041 |  | -0.050 |  | 0.111 |  | 721.814 |  | 0.753 |  | 0.452 |  |
| Volatility1 ✻ AQ ✻ TimeBin3 |  | 2 - 1 ✻ AQ ✻ 4 - 1 |  | 0.040 |  | 0.041 |  | -0.040 |  | 0.121 |  | 721.814 |  | 0.979 |  | 0.328 |  |
| Volatility1 ✻ AQ ✻ TimeBin4 |  | 2 - 1 ✻ AQ ✻ 5 - 1 |  | 0.032 |  | 0.041 |  | -0.049 |  | 0.112 |  | 721.814 |  | 0.771 |  | 0.441 |  |
| Volatility1 ✻ Variability1 ✻ AQ |  | 2 - 1 ✻ 2 - 1 ✻ AQ |  | 0.010 |  | 0.026 |  | -0.041 |  | 0.061 |  | 722.043 |  | 0.396 |  | 0.692 |  |
| Volatility1 ✻ Variability1 ✻ AQ ✻ TimeBin1 |  | 2 - 1 ✻ 2 - 1 ✻ AQ ✻ 2 - 1 |  | 0.034 |  | 0.082 |  | -0.127 |  | 0.195 |  | 721.814 |  | 0.418 |  | 0.676 |  |
| Volatility1 ✻ Variability1 ✻ AQ ✻ TimeBin2 |  | 2 - 1 ✻ 2 - 1 ✻ AQ ✻ 3 - 1 |  | 0.003 |  | 0.082 |  | -0.158 |  | 0.164 |  | 721.814 |  | 0.037 |  | 0.970 |  |
| Volatility1 ✻ Variability1 ✻ AQ ✻ TimeBin3 |  | 2 - 1 ✻ 2 - 1 ✻ AQ ✻ 4 - 1 |  | -0.012 |  | 0.082 |  | -0.173 |  | 0.149 |  | 721.814 |  | -0.149 |  | 0.882 |  |
| Volatility1 ✻ Variability1 ✻ AQ ✻ TimeBin4 |  | 2 - 1 ✻ 2 - 1 ✻ AQ ✻ 5 - 1 |  | 0.017 |  | 0.082 |  | -0.144 |  | 0.178 |  | 721.814 |  | 0.212 |  | 0.832 |  |
|  | | | | | | | | | | | | | | | | | |

| Random Components | | | | | | | | | |
| --- | --- | --- | --- | --- | --- | --- | --- | --- | --- |
|  |  |  |  |  |  |  |  |  |  |
| **Groups** | | **Name** | | **SD** | | **Variance** | | **ICC** | |
| id |  | (Intercept) |  | 0.216 |  | 0.047 |  | 0.039 |  |
| Residual |  |  |  | 1.068 |  | 1.142 |  |  |  |
|  | | | | | | | | | |

Post Hoc Tests

| Post Hoc Comparisons - Variability | | | | | | | | | | | | | | | |
| --- | --- | --- | --- | --- | --- | --- | --- | --- | --- | --- | --- | --- | --- | --- | --- |
| **Comparison** | | | | | |  | | | | | | | | | |
| **Variability** | |  | | **Variability** | | **Difference** | | **SE** | | **t** | | **df** | | **p_bonferroni_** | |
| 1 |  | - |  | 2 |  | -0.162 |  | 0.082 |  | -1.975 |  | 662.208 |  | 0.049 |  |
|  | | | | | | | | | | | | | | | |

## Hypothesis Switch ERPE Mixed Model

Mixed Model

| Model Info | | | |
| --- | --- | --- | --- |
|  |  |  |  |
| **Info** | |  | |
| Estimate |  | Linear mixed model fit by REML |  |
| Call |  | HypSwitchERPE ~ 1 + Variability + Volatility + TimeBin + AQ + avPE + nHypSwitch + Variability:Volatility + Variability:TimeBin + Volatility:TimeBin + Variability:AQ + Volatility:AQ + TimeBin:AQ + Variability:Volatility:TimeBin + Variability:Volatility:AQ + Variability:TimeBin:AQ + Volatility:TimeBin:AQ + Variability:Volatility:TimeBin:AQ+( 1 \| id ) |  |
| AIC |  | 2893.939 |  |
| R-squared Marginal |  | 0.920 |  |
| R-squared Conditional |  | 0.964 |  |
|  | | | |

Model Results

| Fixed Effect Omnibus tests | | | | | | | | | |
| --- | --- | --- | --- | --- | --- | --- | --- | --- | --- |
|  |  |  |  |  |  |  |  |  |  |
|  | | **F** | | **Num df** | | **Den df** | | **p** | |
| Variability |  | 125.092 |  | 1 |  | 511.660 |  | < .001 |  |
| Volatility |  | 6.140 |  | 1 |  | 719.046 |  | 0.013 |  |
| TimeBin |  | 252.294 |  | 4 |  | 718.606 |  | < .001 |  |
| AQ |  | 0.234 |  | 1 |  | 36.500 |  | 0.632 |  |
| avPE |  | 1139.785 |  | 1 |  | 486.130 |  | < .001 |  |
| nHypSwitch |  | 8.626 |  | 1 |  | 271.145 |  | 0.004 |  |
| Variability ✻ Volatility |  | 10.758 |  | 1 |  | 728.613 |  | 0.001 |  |
| Variability ✻ TimeBin |  | 17.938 |  | 4 |  | 718.606 |  | < .001 |  |
| Volatility ✻ TimeBin |  | 0.060 |  | 4 |  | 718.606 |  | 0.993 |  |
| Variability ✻ AQ |  | 3.443 |  | 1 |  | 734.027 |  | 0.064 |  |
| Volatility ✻ AQ |  | 7.034e-6 |  | 1 |  | 718.910 |  | 0.998 |  |
| TimeBin ✻ AQ |  | 12.162 |  | 4 |  | 718.606 |  | < .001 |  |
| Variability ✻ Volatility ✻ TimeBin |  | 0.445 |  | 4 |  | 718.606 |  | 0.776 |  |
| Variability ✻ Volatility ✻ AQ |  | 0.005 |  | 1 |  | 718.957 |  | 0.943 |  |
| Variability ✻ TimeBin ✻ AQ |  | 0.138 |  | 4 |  | 718.606 |  | 0.968 |  |
| Volatility ✻ TimeBin ✻ AQ |  | 0.032 |  | 4 |  | 718.606 |  | 0.998 |  |
| Variability ✻ Volatility ✻ TimeBin ✻ AQ |  | 0.426 |  | 4 |  | 718.606 |  | 0.790 |  |
|  | | | | | | | | | |

| Fixed Effects Parameter Estimates | | | | | | | | | | | | | | | | | |
| --- | --- | --- | --- | --- | --- | --- | --- | --- | --- | --- | --- | --- | --- | --- | --- | --- | --- |
|  | | | | | | | | **95% Confidence Interval** | | | |  | | | | | |
| **Names** | | **Effect** | | **Estimate** | | **SE** | | **Lower** | | **Upper** | | **df** | | **t** | | **p** | |
| (Intercept) |  | (Intercept) |  | 12.384 |  | 0.232 |  | 11.930 |  | 12.838 |  | 35.472 |  | 53.485 |  | < .001 |  |
| Variability1 |  | 2 - 1 |  | -2.386 |  | 0.213 |  | -2.804 |  | -1.968 |  | 511.660 |  | -11.184 |  | < .001 |  |
| Volatility1 |  | 2 - 1 |  | 0.226 |  | 0.091 |  | 0.047 |  | 0.405 |  | 719.046 |  | 2.478 |  | 0.013 |  |
| TimeBin1 |  | 2 - 1 |  | 0.747 |  | 0.144 |  | 0.465 |  | 1.029 |  | 718.606 |  | 5.184 |  | < .001 |  |
| TimeBin2 |  | 3 - 1 |  | 3.655 |  | 0.144 |  | 3.372 |  | 3.937 |  | 718.606 |  | 25.360 |  | < .001 |  |
| TimeBin3 |  | 4 - 1 |  | -0.006 |  | 0.144 |  | -0.289 |  | 0.276 |  | 718.606 |  | -0.043 |  | 0.966 |  |
| TimeBin4 |  | 5 - 1 |  | -0.221 |  | 0.144 |  | -0.504 |  | 0.061 |  | 718.606 |  | -1.537 |  | 0.125 |  |
| AQ |  | AQ |  | -0.019 |  | 0.040 |  | -0.098 |  | 0.059 |  | 36.500 |  | -0.484 |  | 0.632 |  |
| avPE |  | avPE |  | 1.318 |  | 0.039 |  | 1.241 |  | 1.395 |  | 486.130 |  | 33.761 |  | < .001 |  |
| nHypSwitch |  | nHypSwitch |  | -0.049 |  | 0.017 |  | -0.081 |  | -0.016 |  | 271.145 |  | -2.937 |  | 0.004 |  |
| Variability1 ✻ Volatility1 |  | 2 - 1 ✻ 2 - 1 |  | -0.603 |  | 0.184 |  | -0.963 |  | -0.243 |  | 728.613 |  | -3.280 |  | 0.001 |  |
| Variability1 ✻ TimeBin1 |  | 2 - 1 ✻ 2 - 1 |  | -0.467 |  | 0.288 |  | -1.032 |  | 0.098 |  | 718.606 |  | -1.620 |  | 0.106 |  |
| Variability1 ✻ TimeBin2 |  | 2 - 1 ✻ 3 - 1 |  | -2.158 |  | 0.288 |  | -2.723 |  | -1.593 |  | 718.606 |  | -7.488 |  | < .001 |  |
| Variability1 ✻ TimeBin3 |  | 2 - 1 ✻ 4 - 1 |  | -0.433 |  | 0.288 |  | -0.997 |  | 0.132 |  | 718.606 |  | -1.501 |  | 0.134 |  |
| Variability1 ✻ TimeBin4 |  | 2 - 1 ✻ 5 - 1 |  | -0.200 |  | 0.288 |  | -0.765 |  | 0.364 |  | 718.606 |  | -0.695 |  | 0.487 |  |
| Volatility1 ✻ TimeBin1 |  | 2 - 1 ✻ 2 - 1 |  | 0.044 |  | 0.288 |  | -0.521 |  | 0.609 |  | 718.606 |  | 0.153 |  | 0.879 |  |
| Volatility1 ✻ TimeBin2 |  | 2 - 1 ✻ 3 - 1 |  | 0.133 |  | 0.288 |  | -0.432 |  | 0.697 |  | 718.606 |  | 0.460 |  | 0.646 |  |
| Volatility1 ✻ TimeBin3 |  | 2 - 1 ✻ 4 - 1 |  | 0.052 |  | 0.288 |  | -0.513 |  | 0.617 |  | 718.606 |  | 0.181 |  | 0.857 |  |
| Volatility1 ✻ TimeBin4 |  | 2 - 1 ✻ 5 - 1 |  | 0.024 |  | 0.288 |  | -0.541 |  | 0.589 |  | 718.606 |  | 0.083 |  | 0.934 |  |
| Variability1 ✻ AQ |  | 2 - 1 ✻ AQ |  | 0.030 |  | 0.016 |  | -0.002 |  | 0.062 |  | 734.027 |  | 1.855 |  | 0.064 |  |
| Volatility1 ✻ AQ |  | 2 - 1 ✻ AQ |  | -4.157e−5 |  | 0.016 |  | -0.031 |  | 0.031 |  | 718.910 |  | -0.003 |  | 0.998 |  |
| TimeBin1 ✻ AQ |  | 2 - 1 ✻ AQ |  | -0.031 |  | 0.025 |  | -0.080 |  | 0.017 |  | 718.606 |  | -1.268 |  | 0.205 |  |
| TimeBin2 ✻ AQ |  | 3 - 1 ✻ AQ |  | -0.144 |  | 0.025 |  | -0.192 |  | -0.095 |  | 718.606 |  | -5.804 |  | < .001 |  |
| TimeBin3 ✻ AQ |  | 4 - 1 ✻ AQ |  | -0.009 |  | 0.025 |  | -0.058 |  | 0.039 |  | 718.606 |  | -0.381 |  | 0.703 |  |
| TimeBin4 ✻ AQ |  | 5 - 1 ✻ AQ |  | -2.090e−5 |  | 0.025 |  | -0.049 |  | 0.049 |  | 718.606 |  | -8.436e−4 |  | 0.999 |  |
| Variability1 ✻ Volatility1 ✻ TimeBin1 |  | 2 - 1 ✻ 2 - 1 ✻ 2 - 1 |  | -0.217 |  | 0.576 |  | -1.347 |  | 0.912 |  | 718.606 |  | -0.377 |  | 0.706 |  |
| Variability1 ✻ Volatility1 ✻ TimeBin2 |  | 2 - 1 ✻ 2 - 1 ✻ 3 - 1 |  | -0.707 |  | 0.576 |  | -1.837 |  | 0.422 |  | 718.606 |  | -1.227 |  | 0.220 |  |
| Variability1 ✻ Volatility1 ✻ TimeBin3 |  | 2 - 1 ✻ 2 - 1 ✻ 4 - 1 |  | -0.420 |  | 0.576 |  | -1.549 |  | 0.710 |  | 718.606 |  | -0.728 |  | 0.467 |  |
| Variability1 ✻ Volatility1 ✻ TimeBin4 |  | 2 - 1 ✻ 2 - 1 ✻ 5 - 1 |  | -0.164 |  | 0.576 |  | -1.294 |  | 0.966 |  | 718.606 |  | -0.284 |  | 0.776 |  |
| Variability1 ✻ Volatility1 ✻ AQ |  | 2 - 1 ✻ 2 - 1 ✻ AQ |  | 0.002 |  | 0.031 |  | -0.059 |  | 0.064 |  | 718.957 |  | 0.071 |  | 0.943 |  |
| Variability1 ✻ TimeBin1 ✻ AQ |  | 2 - 1 ✻ 2 - 1 ✻ AQ |  | -0.017 |  | 0.050 |  | -0.114 |  | 0.080 |  | 718.606 |  | -0.347 |  | 0.728 |  |
| Variability1 ✻ TimeBin2 ✻ AQ |  | 2 - 1 ✻ 3 - 1 ✻ AQ |  | 0.019 |  | 0.050 |  | -0.079 |  | 0.116 |  | 718.606 |  | 0.374 |  | 0.708 |  |
| Variability1 ✻ TimeBin3 ✻ AQ |  | 2 - 1 ✻ 4 - 1 ✻ AQ |  | 0.002 |  | 0.050 |  | -0.095 |  | 0.099 |  | 718.606 |  | 0.033 |  | 0.974 |  |
| Variability1 ✻ TimeBin4 ✻ AQ |  | 2 - 1 ✻ 5 - 1 ✻ AQ |  | -0.006 |  | 0.050 |  | -0.103 |  | 0.091 |  | 718.606 |  | -0.120 |  | 0.905 |  |
| Volatility1 ✻ TimeBin1 ✻ AQ |  | 2 - 1 ✻ 2 - 1 ✻ AQ |  | -0.007 |  | 0.050 |  | -0.104 |  | 0.090 |  | 718.606 |  | -0.147 |  | 0.883 |  |
| Volatility1 ✻ TimeBin2 ✻ AQ |  | 2 - 1 ✻ 3 - 1 ✻ AQ |  | -0.011 |  | 0.050 |  | -0.109 |  | 0.086 |  | 718.606 |  | -0.232 |  | 0.817 |  |
| Volatility1 ✻ TimeBin3 ✻ AQ |  | 2 - 1 ✻ 4 - 1 ✻ AQ |  | -0.017 |  | 0.050 |  | -0.114 |  | 0.080 |  | 718.606 |  | -0.343 |  | 0.732 |  |
| Volatility1 ✻ TimeBin4 ✻ AQ |  | 2 - 1 ✻ 5 - 1 ✻ AQ |  | -0.012 |  | 0.050 |  | -0.109 |  | 0.086 |  | 718.606 |  | -0.233 |  | 0.816 |  |
| Variability1 ✻ Volatility1 ✻ TimeBin1 ✻ AQ |  | 2 - 1 ✻ 2 - 1 ✻ 2 - 1 ✻ AQ |  | 0.064 |  | 0.099 |  | -0.130 |  | 0.258 |  | 718.606 |  | 0.643 |  | 0.520 |  |
| Variability1 ✻ Volatility1 ✻ TimeBin2 ✻ AQ |  | 2 - 1 ✻ 2 - 1 ✻ 3 - 1 ✻ AQ |  | 0.126 |  | 0.099 |  | -0.068 |  | 0.320 |  | 718.606 |  | 1.271 |  | 0.204 |  |
| Variability1 ✻ Volatility1 ✻ TimeBin3 ✻ AQ |  | 2 - 1 ✻ 2 - 1 ✻ 4 - 1 ✻ AQ |  | 0.071 |  | 0.099 |  | -0.123 |  | 0.265 |  | 718.606 |  | 0.716 |  | 0.474 |  |
| Variability1 ✻ Volatility1 ✻ TimeBin4 ✻ AQ |  | 2 - 1 ✻ 2 - 1 ✻ 5 - 1 ✻ AQ |  | 0.043 |  | 0.099 |  | -0.151 |  | 0.237 |  | 718.606 |  | 0.436 |  | 0.663 |  |
|  | | | | | | | | | | | | | | | | | |

| Random Components | | | | | | | | | |
| --- | --- | --- | --- | --- | --- | --- | --- | --- | --- |
|  |  |  |  |  |  |  |  |  |  |
| **Groups** | | **Name** | | **SD** | | **Variance** | | **ICC** | |
| id |  | (Intercept) |  | 1.436 |  | 2.062 |  | 0.554 |  |
| Residual |  |  |  | 1.289 |  | 1.661 |  |  |  |
|  | | | | | | | | | |

Post Hoc Tests

| Post Hoc Comparisons - Variability | | | | | | | | | | | | | | | |
| --- | --- | --- | --- | --- | --- | --- | --- | --- | --- | --- | --- | --- | --- | --- | --- |
| **Comparison** | | | | | |  | | | | | | | | | |
| **Variability** | |  | | **Variability** | | **Difference** | | **SE** | | **t** | | **df** | | **p_bonferroni_** | |
| 1 |  | - |  | 2 |  | 2.386 |  | 0.215 |  | 11.094 |  | 518.881 |  | < .001 |  |
|  | | | | | | | | | | | | | | | |

| Post Hoc Comparisons - Volatility | | | | | | | | | | | | | | | |
| --- | --- | --- | --- | --- | --- | --- | --- | --- | --- | --- | --- | --- | --- | --- | --- |
| **Comparison** | | | | | |  | | | | | | | | | |
| **Volatility** | |  | | **Volatility** | | **Difference** | | **SE** | | **t** | | **df** | | **p_bonferroni_** | |
| 1 |  | - |  | 2 |  | -0.226 |  | 0.091 |  | -2.478 |  | 720.631 |  | 0.013 |  |
|  | | | | | | | | | | | | | | | |

| Post Hoc Comparisons - TimeBin | | | | | | | | | | | | | | | |
| --- | --- | --- | --- | --- | --- | --- | --- | --- | --- | --- | --- | --- | --- | --- | --- |
| **Comparison** | | | | | |  | | | | | | | | | |
| **TimeBin** | |  | | **TimeBin** | | **Difference** | | **SE** | | **t** | | **df** | | **p_bonferroni_** | |
| 2 |  | - |  | 3 |  | -2.908 |  | 0.144 |  | -20.176 |  | 720.208 |  | < .001 |  |
| 2 |  | - |  | 4 |  | 0.753 |  | 0.144 |  | 5.227 |  | 720.208 |  | < .001 |  |
| 2 |  | - |  | 5 |  | 0.968 |  | 0.144 |  | 6.721 |  | 720.208 |  | < .001 |  |
| 1 |  | - |  | 2 |  | -0.747 |  | 0.144 |  | -5.184 |  | 720.208 |  | < .001 |  |
| 1 |  | - |  | 3 |  | -3.655 |  | 0.144 |  | -25.360 |  | 720.208 |  | < .001 |  |
| 1 |  | - |  | 4 |  | 0.006 |  | 0.144 |  | 0.043 |  | 720.208 |  | 1.000 |  |
| 1 |  | - |  | 5 |  | 0.221 |  | 0.144 |  | 1.537 |  | 720.208 |  | 1.000 |  |
| 3 |  | - |  | 4 |  | 3.661 |  | 0.144 |  | 25.403 |  | 720.208 |  | < .001 |  |
| 3 |  | - |  | 5 |  | 3.876 |  | 0.144 |  | 26.897 |  | 720.208 |  | < .001 |  |
| 4 |  | - |  | 5 |  | 0.215 |  | 0.144 |  | 1.494 |  | 720.208 |  | 1.000 |  |
|  | | | | | | | | | | | | | | | |

| Post Hoc Comparisons - Variability ✻ Volatility | | | | | | | | | | | | | | | | | | | |
| --- | --- | --- | --- | --- | --- | --- | --- | --- | --- | --- | --- | --- | --- | --- | --- | --- | --- | --- | --- |
| **Comparison** | | | | | | | | | |  | | | | | | | | | |
| **Variability** | | **Volatility** | |  | | **Variability** | | **Volatility** | | **Difference** | | **SE** | | **t** | | **df** | | **p_bonferroni_** | |
| 2 |  | 1 |  | - |  | 2 |  | 2 |  | 0.076 |  | 0.129 |  | 0.584 |  | 723.906 |  | 1.000 |  |
| 2 |  | 1 |  | - |  | 1 |  | 2 |  | -2.612 |  | 0.233 |  | -11.218 |  | 584.968 |  | < .001 |  |
| 1 |  | 2 |  | - |  | 2 |  | 2 |  | 2.687 |  | 0.239 |  | 11.223 |  | 565.458 |  | < .001 |  |
| 1 |  | 1 |  | - |  | 2 |  | 2 |  | 2.160 |  | 0.234 |  | 9.217 |  | 589.917 |  | < .001 |  |
| 1 |  | 1 |  | - |  | 2 |  | 1 |  | 2.084 |  | 0.228 |  | 9.135 |  | 608.443 |  | < .001 |  |
| 1 |  | 1 |  | - |  | 1 |  | 2 |  | -0.527 |  | 0.130 |  | -4.068 |  | 726.869 |  | < .001 |  |
|  | | | | | | | | | | | | | | | | | | | |

| Post Hoc Comparisons - Variability ✻ TimeBin | | | | | | | | | | | | | | | | | | | |
| --- | --- | --- | --- | --- | --- | --- | --- | --- | --- | --- | --- | --- | --- | --- | --- | --- | --- | --- | --- |
| **Comparison** | | | | | | | | | |  | | | | | | | | | |
| **Variability** | | **TimeBin** | |  | | **Variability** | | **TimeBin** | | **Difference** | | **SE** | | **t** | | **df** | | **p_bonferroni_** | |
| 2 |  | 2 |  | - |  | 2 |  | 3 |  | -2.062 |  | 0.204 |  | -10.117 |  | 720.208 |  | < .001 |  |
| 2 |  | 2 |  | - |  | 2 |  | 4 |  | 0.736 |  | 0.204 |  | 3.612 |  | 720.208 |  | 0.015 |  |
| 2 |  | 2 |  | - |  | 2 |  | 5 |  | 0.835 |  | 0.204 |  | 4.099 |  | 720.208 |  | 0.002 |  |
| 2 |  | 2 |  | - |  | 1 |  | 3 |  | -5.954 |  | 0.282 |  | -21.121 |  | 697.056 |  | < .001 |  |
| 2 |  | 2 |  | - |  | 1 |  | 4 |  | -1.431 |  | 0.282 |  | -5.075 |  | 697.056 |  | < .001 |  |
| 2 |  | 2 |  | - |  | 1 |  | 5 |  | -1.099 |  | 0.282 |  | -3.900 |  | 697.056 |  | 0.005 |  |
| 2 |  | 1 |  | - |  | 2 |  | 2 |  | -0.514 |  | 0.204 |  | -2.520 |  | 720.208 |  | 0.537 |  |
| 2 |  | 1 |  | - |  | 2 |  | 3 |  | -2.575 |  | 0.204 |  | -12.638 |  | 720.208 |  | < .001 |  |
| 2 |  | 1 |  | - |  | 2 |  | 4 |  | 0.222 |  | 0.204 |  | 1.091 |  | 720.208 |  | 1.000 |  |
| 2 |  | 1 |  | - |  | 2 |  | 5 |  | 0.322 |  | 0.204 |  | 1.578 |  | 720.208 |  | 1.000 |  |
| 2 |  | 1 |  | - |  | 1 |  | 2 |  | -2.715 |  | 0.282 |  | -9.630 |  | 697.056 |  | < .001 |  |
| 2 |  | 1 |  | - |  | 1 |  | 3 |  | -6.468 |  | 0.282 |  | -22.943 |  | 697.056 |  | < .001 |  |
| 2 |  | 1 |  | - |  | 1 |  | 4 |  | -1.944 |  | 0.282 |  | -6.897 |  | 697.056 |  | < .001 |  |
| 2 |  | 1 |  | - |  | 1 |  | 5 |  | -1.613 |  | 0.282 |  | -5.722 |  | 697.056 |  | < .001 |  |
| 2 |  | 3 |  | - |  | 2 |  | 4 |  | 2.798 |  | 0.204 |  | 13.729 |  | 720.208 |  | < .001 |  |
| 2 |  | 3 |  | - |  | 2 |  | 5 |  | 2.897 |  | 0.204 |  | 14.216 |  | 720.208 |  | < .001 |  |
| 2 |  | 3 |  | - |  | 1 |  | 4 |  | 0.631 |  | 0.282 |  | 2.239 |  | 697.056 |  | 1.000 |  |
| 2 |  | 3 |  | - |  | 1 |  | 5 |  | 0.962 |  | 0.282 |  | 3.414 |  | 697.056 |  | 0.030 |  |
| 2 |  | 4 |  | - |  | 2 |  | 5 |  | 0.099 |  | 0.204 |  | 0.487 |  | 720.208 |  | 1.000 |  |
| 2 |  | 4 |  | - |  | 1 |  | 5 |  | -1.835 |  | 0.282 |  | -6.511 |  | 697.056 |  | < .001 |  |
| 1 |  | 2 |  | - |  | 2 |  | 2 |  | 2.201 |  | 0.282 |  | 7.808 |  | 697.056 |  | < .001 |  |
| 1 |  | 2 |  | - |  | 2 |  | 3 |  | 0.139 |  | 0.282 |  | 0.494 |  | 697.056 |  | 1.000 |  |
| 1 |  | 2 |  | - |  | 2 |  | 4 |  | 2.937 |  | 0.282 |  | 10.419 |  | 697.056 |  | < .001 |  |
| 1 |  | 2 |  | - |  | 2 |  | 5 |  | 3.036 |  | 0.282 |  | 10.771 |  | 697.056 |  | < .001 |  |
| 1 |  | 2 |  | - |  | 1 |  | 3 |  | -3.753 |  | 0.204 |  | -18.416 |  | 720.208 |  | < .001 |  |
| 1 |  | 2 |  | - |  | 1 |  | 4 |  | 0.770 |  | 0.204 |  | 3.780 |  | 720.208 |  | 0.008 |  |
| 1 |  | 2 |  | - |  | 1 |  | 5 |  | 1.102 |  | 0.204 |  | 5.406 |  | 720.208 |  | < .001 |  |
| 1 |  | 1 |  | - |  | 2 |  | 2 |  | 1.221 |  | 0.282 |  | 4.330 |  | 697.056 |  | < .001 |  |
| 1 |  | 1 |  | - |  | 2 |  | 1 |  | 1.734 |  | 0.282 |  | 6.152 |  | 697.056 |  | < .001 |  |
| 1 |  | 1 |  | - |  | 2 |  | 3 |  | -0.841 |  | 0.282 |  | -2.984 |  | 697.056 |  | 0.132 |  |
| 1 |  | 1 |  | - |  | 2 |  | 4 |  | 1.957 |  | 0.282 |  | 6.941 |  | 697.056 |  | < .001 |  |
| 1 |  | 1 |  | - |  | 2 |  | 5 |  | 2.056 |  | 0.282 |  | 7.293 |  | 697.056 |  | < .001 |  |
| 1 |  | 1 |  | - |  | 1 |  | 2 |  | -0.980 |  | 0.204 |  | -4.811 |  | 720.208 |  | < .001 |  |
| 1 |  | 1 |  | - |  | 1 |  | 3 |  | -4.734 |  | 0.204 |  | -23.227 |  | 720.208 |  | < .001 |  |
| 1 |  | 1 |  | - |  | 1 |  | 4 |  | -0.210 |  | 0.204 |  | -1.031 |  | 720.208 |  | 1.000 |  |
| 1 |  | 1 |  | - |  | 1 |  | 5 |  | 0.121 |  | 0.204 |  | 0.595 |  | 720.208 |  | 1.000 |  |
| 1 |  | 3 |  | - |  | 2 |  | 3 |  | 3.892 |  | 0.282 |  | 13.807 |  | 697.056 |  | < .001 |  |
| 1 |  | 3 |  | - |  | 2 |  | 4 |  | 6.690 |  | 0.282 |  | 23.732 |  | 697.056 |  | < .001 |  |
| 1 |  | 3 |  | - |  | 2 |  | 5 |  | 6.789 |  | 0.282 |  | 24.084 |  | 697.056 |  | < .001 |  |
| 1 |  | 3 |  | - |  | 1 |  | 4 |  | 4.523 |  | 0.204 |  | 22.196 |  | 720.208 |  | < .001 |  |
| 1 |  | 3 |  | - |  | 1 |  | 5 |  | 4.855 |  | 0.204 |  | 23.822 |  | 720.208 |  | < .001 |  |
| 1 |  | 4 |  | - |  | 2 |  | 4 |  | 2.167 |  | 0.282 |  | 7.686 |  | 697.056 |  | < .001 |  |
| 1 |  | 4 |  | - |  | 2 |  | 5 |  | 2.266 |  | 0.282 |  | 8.038 |  | 697.056 |  | < .001 |  |
| 1 |  | 4 |  | - |  | 1 |  | 5 |  | 0.331 |  | 0.204 |  | 1.626 |  | 720.208 |  | 1.000 |  |
| 1 |  | 5 |  | - |  | 2 |  | 5 |  | 1.935 |  | 0.282 |  | 6.863 |  | 697.056 |  | < .001 |  |
|  | | | | | | | | | | | | | | | | | | | |

Simple Effects

| Simple effects of AQ : Omnibus Tests | | | | | | | | | |
| --- | --- | --- | --- | --- | --- | --- | --- | --- | --- |
| **Moderator levels** | |  | | | | | | | |
| **TimeBin** | | **F** | | **Num df** | | **Den df** | | **p** | |
| 1 |  | 0.165 |  | 1.000 |  | 50.450 |  | 0.686 |  |
| 2 |  | 0.104 |  | 1.000 |  | 50.450 |  | 0.748 |  |
| 3 |  | 8.576 |  | 1.000 |  | 50.450 |  | 0.005 |  |
| 4 |  | 0.035 |  | 1.000 |  | 50.450 |  | 0.852 |  |
| 5 |  | 0.165 |  | 1.000 |  | 50.450 |  | 0.687 |  |
|  | | | | | | | | | |

| Simple effects of AQ : Parameter estimates | | | | | | | | | | | | | | | |
| --- | --- | --- | --- | --- | --- | --- | --- | --- | --- | --- | --- | --- | --- | --- | --- |
| **Moderator levels** | |  | | | | **95% Confidence Interval** | | | |  | | | | | |
| **TimeBin** | | **Estimate** | | **SE** | | **Lower** | | **Upper** | | **df** | | **t** | | **p** | |
| 1 |  | 0.018 |  | 0.043 |  | -0.069 |  | 0.104 |  | 50.450 |  | 0.406 |  | 0.686 |  |
| 2 |  | -0.014 |  | 0.043 |  | -0.100 |  | 0.073 |  | 50.450 |  | -0.322 |  | 0.748 |  |
| 3 |  | -0.126 |  | 0.043 |  | -0.213 |  | -0.040 |  | 50.450 |  | -2.928 |  | 0.005 |  |
| 4 |  | 0.008 |  | 0.043 |  | -0.079 |  | 0.095 |  | 50.450 |  | 0.187 |  | 0.852 |  |
| 5 |  | 0.017 |  | 0.043 |  | -0.069 |  | 0.104 |  | 50.450 |  | 0.406 |  | 0.687 |  |
| Note. Simple effects are estimated setting higher order moderator (if any) in covariates to zero and averaging across moderating factors levels (if any) | | | | | | | | | | | | | | | |
|  | | | | | | | | | | | | | | | |

Correlation Matrix

| Correlation Matrix | | | | | | | |
| --- | --- | --- | --- | --- | --- | --- | --- |
|  |  |  |  |  |  |  |  |
|  | |  | | **AQ** | | **HypSwitchERPE** | |
| AQ |  | Pearson's r |  | — |  |  |  |
|  |  | p-value |  | — |  |  |  |
| HypSwitchERPE |  | Pearson's r |  | -0.211 |  | — |  |
|  |  | p-value |  | < .001 |  | — |  |
|  | | | | | | | |

Plot


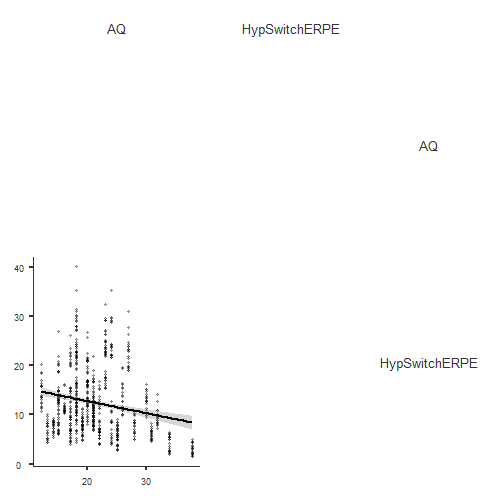


## Incorrect Trials Only Hypothesis Switch ERPE Mixed Model

Mixed Model

| Model Info | | | |
| --- | --- | --- | --- |
|  |  |  |  |
| **Info** | |  | |
| Estimate |  | Linear mixed model fit by REML |  |
| Call |  | HypSwitchIncorrectERPE ~ 1 + Variability + Volatility + AQ + TimeBin + nHypSwitch + avPE + Variability:Volatility + Variability:TimeBin + Volatility:TimeBin + Variability:Volatility:TimeBin+( 1 \| id ) |  |
| AIC |  | 4222.870 |  |
| R-squared Marginal |  | 0.697 |  |
| R-squared Conditional |  | 0.800 |  |
|  | | | |

Model Results

| Fixed Effect Omnibus tests | | | | | | | | | |
| --- | --- | --- | --- | --- | --- | --- | --- | --- | --- |
|  |  |  |  |  |  |  |  |  |  |
|  | | **F** | | **Num df** | | **Den df** | | **p** | |
| Variability |  | 44.1261 |  | 1 |  | 300.6 |  | < .001 |  |
| Volatility |  | 12.2004 |  | 1 |  | 721.3 |  | < .001 |  |
| AQ |  | 0.8675 |  | 1 |  | 37.2 |  | 0.358 |  |
| TimeBin |  | 22.8229 |  | 4 |  | 718.8 |  | < .001 |  |
| nHypSwitch |  | 2.1231 |  | 1 |  | 132.1 |  | 0.147 |  |
| avPE |  | 213.9828 |  | 1 |  | 236.7 |  | < .001 |  |
| Variability ✻ Volatility |  | 0.0233 |  | 1 |  | 729.1 |  | 0.879 |  |
| Variability ✻ TimeBin |  | 1.8091 |  | 4 |  | 718.8 |  | 0.125 |  |
| Volatility ✻ TimeBin |  | 0.1829 |  | 4 |  | 718.8 |  | 0.947 |  |
| Variability ✻ Volatility ✻ TimeBin |  | 0.1215 |  | 4 |  | 718.8 |  | 0.975 |  |
|  | | | | | | | | | |

| Fixed Effects Parameter Estimates | | | | | | | | | | | | | | | | | |
| --- | --- | --- | --- | --- | --- | --- | --- | --- | --- | --- | --- | --- | --- | --- | --- | --- | --- |
|  | | | | | | | | **95% Confidence Interval** | | | |  | | | | | |
| **Names** | | **Effect** | | **Estimate** | | **SE** | | **Lower** | | **Upper** | | **df** | | **t** | | **p** | |
| (Intercept) |  | (Intercept) |  | 13.6946 |  | 0.3987 |  | 12.913 |  | 14.4761 |  | 35.9 |  | 34.346 |  | < .001 |  |
| Variability1 |  | 2 - 1 |  | -3.2973 |  | 0.4964 |  | -4.270 |  | -2.3244 |  | 300.6 |  | -6.643 |  | < .001 |  |
| Volatility1 |  | 2 - 1 |  | 0.8430 |  | 0.2413 |  | 0.370 |  | 1.3160 |  | 721.3 |  | 3.493 |  | < .001 |  |
| AQ |  | AQ |  | -0.0647 |  | 0.0695 |  | -0.201 |  | 0.0715 |  | 37.2 |  | -0.931 |  | 0.358 |  |
| TimeBin1 |  | 2 - 1 |  | 0.5932 |  | 0.3797 |  | -0.151 |  | 1.3374 |  | 718.8 |  | 1.562 |  | 0.119 |  |
| TimeBin2 |  | 3 - 1 |  | 2.8187 |  | 0.3797 |  | 2.074 |  | 3.5629 |  | 718.8 |  | 7.423 |  | < .001 |  |
| TimeBin3 |  | 4 - 1 |  | -0.1177 |  | 0.3797 |  | -0.862 |  | 0.6265 |  | 718.8 |  | -0.310 |  | 0.757 |  |
| TimeBin4 |  | 5 - 1 |  | -0.2853 |  | 0.3797 |  | -1.030 |  | 0.4589 |  | 718.8 |  | -0.751 |  | 0.453 |  |
| nHypSwitch |  | nHypSwitch |  | -0.0529 |  | 0.0363 |  | -0.124 |  | 0.0182 |  | 132.1 |  | -1.457 |  | 0.147 |  |
| avPE |  | avPE |  | 1.2958 |  | 0.0886 |  | 1.122 |  | 1.4694 |  | 236.7 |  | 14.628 |  | < .001 |  |
| Variability1 ✻ Volatility1 |  | 2 - 1 ✻ 2 - 1 |  | -0.0740 |  | 0.4854 |  | -1.025 |  | 0.8773 |  | 729.1 |  | -0.153 |  | 0.879 |  |
| Variability1 ✻ TimeBin1 |  | 2 - 1 ✻ 2 - 1 |  | -0.5160 |  | 0.7594 |  | -2.004 |  | 0.9725 |  | 718.8 |  | -0.679 |  | 0.497 |  |
| Variability1 ✻ TimeBin2 |  | 2 - 1 ✻ 3 - 1 |  | -1.8104 |  | 0.7594 |  | -3.299 |  | -0.3219 |  | 718.8 |  | -2.384 |  | 0.017 |  |
| Variability1 ✻ TimeBin3 |  | 2 - 1 ✻ 4 - 1 |  | -0.3250 |  | 0.7594 |  | -1.813 |  | 1.1635 |  | 718.8 |  | -0.428 |  | 0.669 |  |
| Variability1 ✻ TimeBin4 |  | 2 - 1 ✻ 5 - 1 |  | -0.1695 |  | 0.7594 |  | -1.658 |  | 1.3190 |  | 718.8 |  | -0.223 |  | 0.823 |  |
| Volatility1 ✻ TimeBin1 |  | 2 - 1 ✻ 2 - 1 |  | 0.3876 |  | 0.7594 |  | -1.101 |  | 1.8761 |  | 718.8 |  | 0.510 |  | 0.610 |  |
| Volatility1 ✻ TimeBin2 |  | 2 - 1 ✻ 3 - 1 |  | 0.6374 |  | 0.7594 |  | -0.851 |  | 2.1259 |  | 718.8 |  | 0.839 |  | 0.402 |  |
| Volatility1 ✻ TimeBin3 |  | 2 - 1 ✻ 4 - 1 |  | 0.3769 |  | 0.7594 |  | -1.112 |  | 1.8654 |  | 718.8 |  | 0.496 |  | 0.620 |  |
| Volatility1 ✻ TimeBin4 |  | 2 - 1 ✻ 5 - 1 |  | 0.4192 |  | 0.7594 |  | -1.069 |  | 1.9076 |  | 718.8 |  | 0.552 |  | 0.581 |  |
| Variability1 ✻ Volatility1 ✻ TimeBin1 |  | 2 - 1 ✻ 2 - 1 ✻ 2 - 1 |  | -0.4618 |  | 1.5189 |  | -3.439 |  | 2.5151 |  | 718.8 |  | -0.304 |  | 0.761 |  |
| Variability1 ✻ Volatility1 ✻ TimeBin2 |  | 2 - 1 ✻ 2 - 1 ✻ 3 - 1 |  | -0.8884 |  | 1.5189 |  | -3.865 |  | 2.0886 |  | 718.8 |  | -0.585 |  | 0.559 |  |
| Variability1 ✻ Volatility1 ✻ TimeBin3 |  | 2 - 1 ✻ 2 - 1 ✻ 4 - 1 |  | -0.7765 |  | 1.5189 |  | -3.753 |  | 2.2004 |  | 718.8 |  | -0.511 |  | 0.609 |  |
| Variability1 ✻ Volatility1 ✻ TimeBin4 |  | 2 - 1 ✻ 2 - 1 ✻ 5 - 1 |  | -0.8593 |  | 1.5189 |  | -3.836 |  | 2.1177 |  | 718.8 |  | -0.566 |  | 0.572 |  |
|  | | | | | | | | | | | | | | | | | |

| Random Components | | | | | | | | | |
| --- | --- | --- | --- | --- | --- | --- | --- | --- | --- |
|  |  |  |  |  |  |  |  |  |  |
| **Groups** | | **Name** | | **SD** | | **Variance** | | **ICC** | |
| id |  | (Intercept) |  | 2.40 |  | 5.77 |  | 0.339 |  |
| Residual |  |  |  | 3.35 |  | 11.23 |  |  |  |
|  | | | | | | | | | |

Post Hoc Tests

| Post Hoc Comparisons - TimeBin | | | | | | | | | | | | | | | |
| --- | --- | --- | --- | --- | --- | --- | --- | --- | --- | --- | --- | --- | --- | --- | --- |
| **Comparison** | | | | | |  | | | | | | | | | |
| **TimeBin** | |  | | **TimeBin** | | **Difference** | | **SE** | | **t** | | **df** | | **p_bonferroni_** | |
| 2 |  | - |  | 3 |  | -2.225 |  | 0.380 |  | -5.861 |  | 720 |  | < .001 |  |
| 2 |  | - |  | 4 |  | 0.711 |  | 0.380 |  | 1.872 |  | 720 |  | 0.616 |  |
| 2 |  | - |  | 5 |  | 0.879 |  | 0.380 |  | 2.314 |  | 720 |  | 0.210 |  |
| 1 |  | - |  | 2 |  | -0.593 |  | 0.380 |  | -1.562 |  | 720 |  | 1.000 |  |
| 1 |  | - |  | 3 |  | -2.819 |  | 0.380 |  | -7.423 |  | 720 |  | < .001 |  |
| 1 |  | - |  | 4 |  | 0.118 |  | 0.380 |  | 0.310 |  | 720 |  | 1.000 |  |
| 1 |  | - |  | 5 |  | 0.285 |  | 0.380 |  | 0.751 |  | 720 |  | 1.000 |  |
| 3 |  | - |  | 4 |  | 2.936 |  | 0.380 |  | 7.733 |  | 720 |  | < .001 |  |
| 3 |  | - |  | 5 |  | 3.104 |  | 0.380 |  | 8.174 |  | 720 |  | < .001 |  |
| 4 |  | - |  | 5 |  | 0.168 |  | 0.380 |  | 0.441 |  | 720 |  | 1.000 |  |
|  | | | | | | | | | | | | | | | |

| Post Hoc Comparisons - Volatility | | | | | | | | | | | | | | | |
| --- | --- | --- | --- | --- | --- | --- | --- | --- | --- | --- | --- | --- | --- | --- | --- |
| **Comparison** | | | | | |  | | | | | | | | | |
| **Volatility** | |  | | **Volatility** | | **Difference** | | **SE** | | **t** | | **df** | | **p_bonferroni_** | |
| 1 |  | - |  | 2 |  | -0.843 |  | 0.241 |  | -3.49 |  | 722 |  | < .001 |  |
|  | | | | | | | | | | | | | | | |

| Post Hoc Comparisons - Variability | | | | | | | | | | | | | | | |
| --- | --- | --- | --- | --- | --- | --- | --- | --- | --- | --- | --- | --- | --- | --- | --- |
| **Comparison** | | | | | |  | | | | | | | | | |
| **Variability** | |  | | **Variability** | | **Difference** | | **SE** | | **t** | | **df** | | **p_bonferroni_** | |
| 1 |  | - |  | 2 |  | 3.30 |  | 0.501 |  | 6.58 |  | 304 |  | < .001 |  |
|  | | | | | | | | | | | | | | | |

## Incorrect Trials Only Hypothesis Switch ERPE Figure


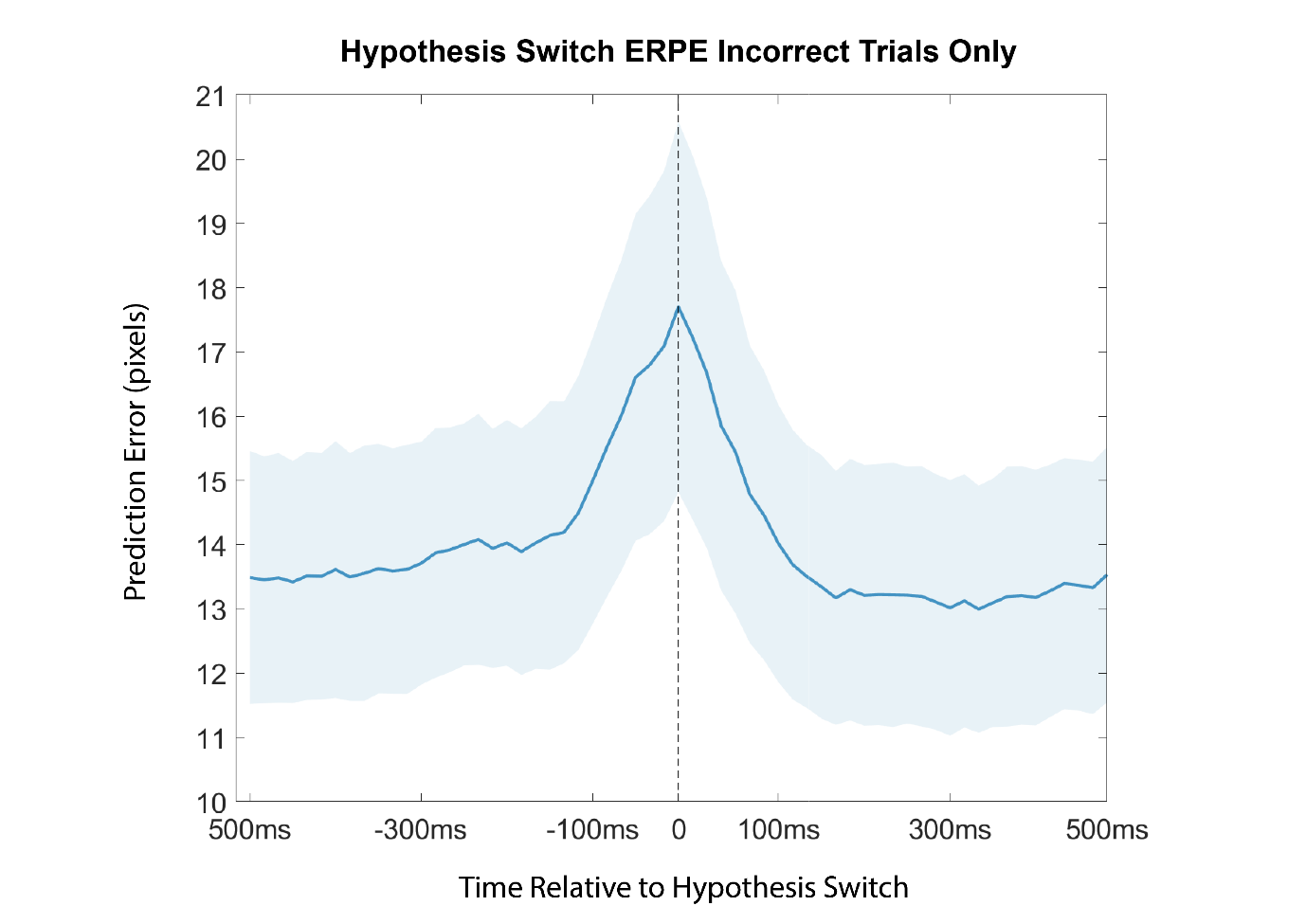


The grand average (blue line) prediction error across participants in a one second epoch centered on hypothesis switches for incorrect trials only. Shading represents a 95% CI.
